# Dectin-1/2–induced autocrine PGE_2_ signaling licenses dendritic cells to prime Th2 responses

**DOI:** 10.1371/journal.pbio.2005504

**Published:** 2018-04-18

**Authors:** Maria M. M. Kaisar, Manuel Ritter, Carlos del Fresno, Hulda S. Jónasdóttir, Alwin J. van der Ham, Leonard R. Pelgrom, Gabriele Schramm, Laura E. Layland, David Sancho, Clarissa Prazeres da Costa, Martin Giera, Maria Yazdanbakhsh, Bart Everts

**Affiliations:** 1 Department of Parasitology, Leiden University Medical Center, Leiden, the Netherlands; 2 Department of Parasitology, Faculty of Medicine, Universitas Indonesia, Jakarta, Indonesia; 3 Institute of Medical Microbiology, Immunology and Parasitology, University Hospital of Bonn, Germany; 4 Centro Nacional de Investigaciones Cardiovasculares “Carlos III”, Madrid, Spain; 5 Center of Proteomics and Metabolomics, Leiden University Medical Center, Leiden, the Netherlands; 6 Research Center Borstel, Borstel, Germany; 7 Institute of Medical Microbiology, Immunology and Parasitology, University Hospital of Bonn, Germany & German Centre for Infection Research, partner site, Bonn-Cologne, Bonn, Germany; 8 Institute for Microbiology, Immunology and Hygiene, Technische Universität München, Germany; New York University, United States of America

## Abstract

The molecular mechanisms through which dendritic cells (DCs) prime T helper 2 (Th2) responses, including those elicited by parasitic helminths, remain incompletely understood. Here, we report that soluble egg antigen (SEA) from *Schistosoma mansoni*, which is well known to drive potent Th2 responses, triggers DCs to produce prostaglandin E2 (PGE_2_), which subsequently—in an autocrine manner—induces OX40 ligand (OX40L) expression to license these DCs to drive Th2 responses. Mechanistically, SEA was found to promote PGE_2_ synthesis through Dectin-1 and Dectin-2, and via a downstream signaling cascade involving spleen tyrosine kinase (Syk), extracellular signal-regulated kinase (ERK), cytosolic phospholipase A_2_ (cPLA_2_), and cyclooxygenase 1 and 2 (COX-1 and COX-2). In addition, this pathway was activated independently of the actions of omega-1 (ω-1), a previously described Th2-priming glycoprotein present in SEA. These findings were supported by in vivo murine data showing that ω-1–independent Th2 priming by SEA was mediated by Dectin-2 and Syk signaling in DCs. Finally, we found that Dectin-2^−/−^, and to a lesser extent Dectin-1^−/−^ mice, displayed impaired Th2 responses and reduced egg-driven granuloma formation following *S*. *mansoni* infection, highlighting the physiological importance of this pathway in Th2 polarization during a helminth infection. In summary, we identified a novel pathway in DCs involving Dectin-1/2-Syk-PGE_2_-OX40L through which Th2 immune responses are induced.

## Author summary

T helper 2 (Th2) responses, which are initiated by dendritic cells (DCs), can cause allergic diseases, but they can also provide protection against metabolic disorders and parasitic helminth infections. As such, there is great interest in better understanding how their activity is induced and regulated by DCs. Parasitic helminths can potently induce Th2 responses. However, how helminths condition DCs for priming of Th2 responses remains incompletely understood. Here, we find that egg antigens from the parasitic helminth *Schistosoma mansoni* bind to pattern-recognition receptors (PRRs) Dectin-1 and Dectin-2 on DCs. This binding triggers a signaling cascade in DCs that results in synthesis of eicosanoid prostaglandin E2 (PGE_2_). PGE_2_ is sensed by the DCs themselves, resulting in expression of OX40 ligand (OX40L), which subsequently enables the DCs to promote Th2 differentiation. We show that this pathway is activated independently of omega-1 (ω-1), which is a glycoprotein secreted by the eggs and previously shown to condition DCs for priming of Th2 responses. Moreover, we demonstrate that this ω-1–independent pathway is crucial for Th2 induction and egg-driven immunopathology following *S*. *mansoni* infection in vivo. In summary, we identified a novel pathway in DCs involving Dectin-1/2–induced autocrine PGE_2_ signaling through which Th2 responses are induced.

## Introduction

Dendritic cells (DCs) are key players in the immune system because of their unique capacity to prime antigen-specific T helper 1 (Th1), Th2, Th17, or regulatory T cell (Treg) responses tailored against the pathogen they encounter [1–3]. It is well known that allergens and parasitic helminths can evoke strong type 2 immune responses, which largely depend on DCs that prime Th2 responses [4–8]. However, the molecular mechanisms through which DCs prime Th2 responses are still not fully defined.

Soluble egg antigen (SEA) from *Schistosoma mansoni* is a widely used antigen mixture to study Th2 responses to helminths. SEA is well recognized for its ability to condition DCs for priming of Th2 responses [9–13]. Omega-1 (ω-1), a glycosylated T2 RNase [14] present in SEA, was found to be a major Th2-polarizing molecule [9,15–18]. Mechanistic studies revealed that ω-1 is bound and internalized via its glycans by the mannose receptor (MR) and that, following uptake, ω-1 impairs protein synthesis in an RNase-dependent manner that is essential for conditioning of DCs for Th2 polarization [9,10]. However, while ω-1 by itself was sufficient to condition DCs for Th2 polarization, SEA from which ω-1 was depleted still retained most of its Th2 priming potential both in vitro and in vivo. Moreover, eggs in which ω-1 expression was silenced [19] retained most of their Th2-polarizing potential, suggesting that there are additional mechanisms through which DCs become conditioned by schistosome eggs to prime Th2 responses [20].

Lipid mediators (LMs)—which arise from the enzymatic oxidation of polyunsaturated fatty acids (PUFAs), such as arachidonic acid (AA), docosahexaenoic acid, or linoleic acid—play an important role in immunological responses. In particular, prostanoids such as tromboxanes and prostaglandins (PGs)—which are derivatives of AA and are primarily released by myeloid cells, including macrophages and DCs—have been shown to have to capacity to influence immune cells by affecting their migration, differentiation, effector function, and/or polarization [21–24]. We recently performed a detailed analysis of the lipidome of the different life stages of *S*. *mansoni* and found that the eggs and egg-derived antigen preparations had a unique lipid profile characterized by the presence of various PGs and leukotrienes [25]. Thus far, efforts to identify molecules responsible for Th2 polarization by helminths have primarily focused on glycans and (glyco)proteins. Whether LMs directly derived from schistosomes, or derived from immune cells in response to infection by this parasite, may additionally affect immune polarization remains unknown.

To identify potential novel pathways through which Th2 responses are induced by *S*. *mansoni*, we set out to assess the role of PUFAs and LMs in *S*. *mansoni* egg-driven Th2 polarization. We here report that SEA, apart from containing various LMs, induces DCs to generate several PGs and leukotrienes, including prostaglandin E2 (PGE_2_), independently of ω-1. We show that this de novo synthesis of PGE_2_ by SEA-stimulated DCs is driven by signaling through Dectin-1 and Dectin-2 and is crucial for Th2 priming. Mechanistically, we provide evidence that this PGE_2_ through autocrine signaling induces OX40 ligand (OX40L) expression, to license DCs to prime Th2 responses. Finally, we show that this pathway is also crucial for Th2 priming by *S*. *mansoni* in vivo.

## Results

### SEA contains PGE_2_ and promotes its synthesis by human monocyte-derived DCs independently of ω-1

**Fig 1:**
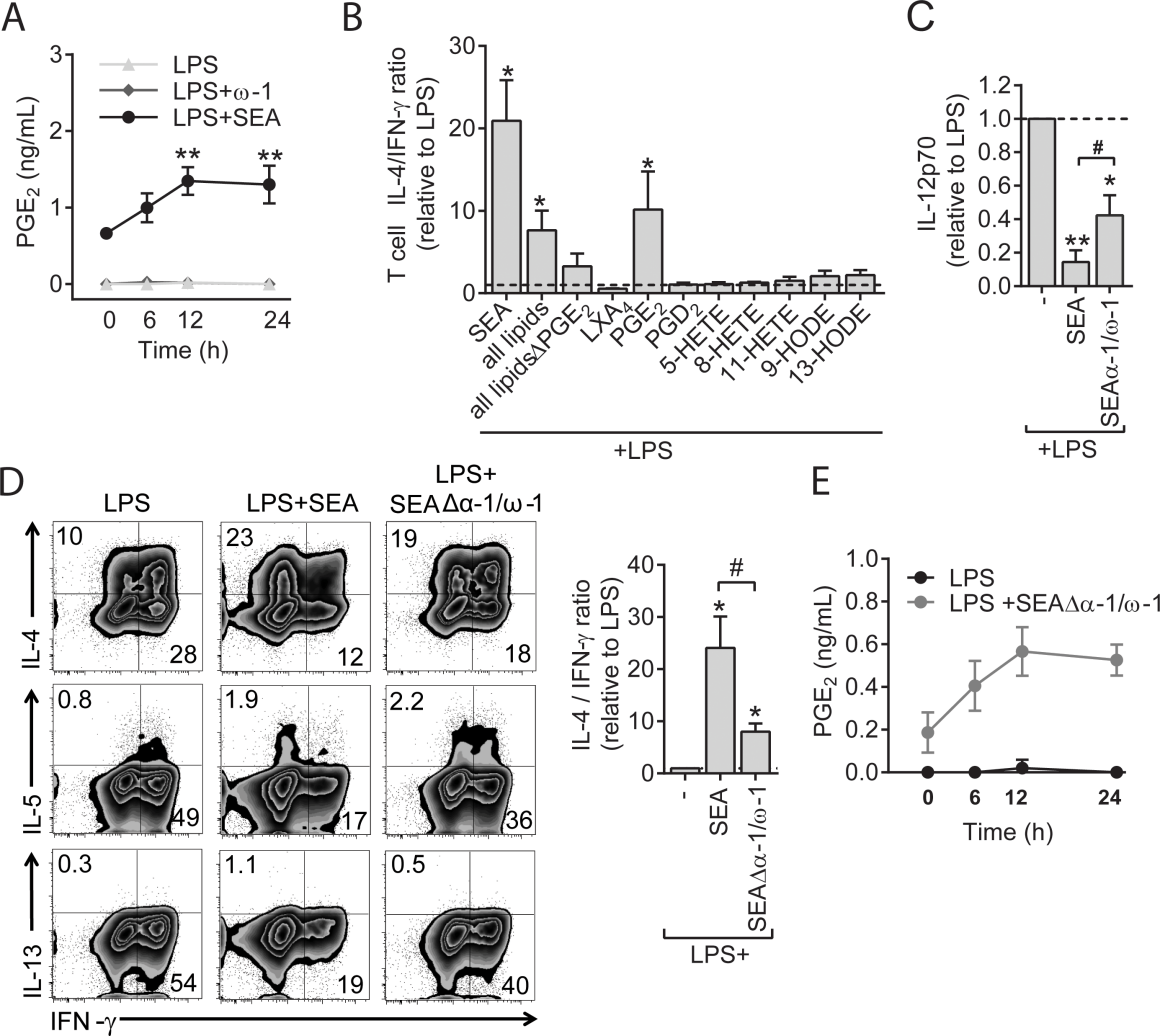
SEA stimulates PGE_2_ secretion and primes Th2 responses independently of ω-1 in human moDCs. (A) PGE_2_ concentration in supernatants from moDC cultures after stimulation with indicated reagents. Concentrations are determined based on an internal standard. Data represent mean ± SEM of 4 independent experiments. (B) moDCs stimulated with indicated lipids (concentration of 2.5 ng/mL for LXA_4_, PGE_2_, and PGD_2_; 12.5 μg/mL for 5-HETE, 8-HETE, and 11-HETE; 25 μg/mL 9-HODE and 13-HODE) were analyzed for Th2 polarizing potential as described in Materials and methods. The ratio of percentage of IL-4^+^ over percentage of IFN-γ^+^ T cells based on intracellular cytokine staining was calculated relative to the control condition. (C) moDCs were pulsed with indicated stimuli and subsequently cocultured with a CD40L-expressing cell line. Supernatants were collected after 24 h, and IL-12p70 concentration was determined by ELISA. (D) T-cell polarization was determined as in panel B. Top and bottom panels show representative flow cytometry plots of intracellular staining of CD4^+^ T cells for indicated cytokines, and the ratio of IL-4 over IFN-γ ratio of these plots based on 4 experiments. Numbers in plots represent frequencies of cells in indicated quadrants. (E) PGE_2_ levels as determined by LC-MS/MS in supernatants of moDCs stimulated with indicated stimuli. Data represent mean ± SEM of 3 independent experiments. (A) Statistical significance of different time points per condition compared to baseline (0 h) time point. “*” and “#”: *P* < 0.05; “**”: *P* < 0.01. (A) based on two-way ANOVA test or (B–D) for significantly different with the LPS control (*) or between-test conditions (#) based on paired analysis (paired Student *t* test). Underlying data can be found in S1 Data. CD4, cluster of differentiation 4; HETE, Hydroxyeicosatetraenoic acid; HODE, Hydroxyoctadecadienoic acid; IFN-γ, interferon γ; IL-4, interleukin 4; LC-MS/MS, liquid chromatography tandem mass spectrometry; LPS, lipopolysaccharide; LXA_4_, lipoxin A_4_; moDC, monocyte-derived DC; PGE_2_, prostaglandin E2; SEA, soluble egg antigen; Th2, T helper 2.

In a recent lipidome analysis, we identified the presence of various LMs in SEA [25]. To explore the immunological significance of these findings, we first quantified these LMs in SEA using a sensitive liquid chromatography tandem mass spectrometry (LC-MS/MS)–based platform. We discovered that SEA contains 22 out of 55 monitored analytes (S1 Table), including docosahexaenoic acid, linoleic acid, and AA in relative high abundance, but also PGs such as PGE_2_ and PGD_2_ (S1 Fig, panel A). To assess potential consumption or uptake of such lipids by DCs, we tested supernatants of monocyte-derived DCs (moDCs) at 0, 6, 12, and 24 h after stimulation with lipopolysaccharide (LPS), LPS plus SEA, and LPS plus ω-1. We observed that levels of the majority of the LMs present in SEA decreased over time in DC culture supernatants following SEA stimulation, indicative of consumption/uptake or degradation (S1 Fig, panel B). We also observed that some of these lipids (i.e., 13-Hydroxyoctadecadienoic acid [13-HODE], lipoxin A_4_ [LXA_4_], PGD_2_, and PGE_2_) were accumulating over time in DC culture supernatants in response to SEA stimulation both in the presence (S1B Fig and quantitated for PGE_2_ in Fig 1A) or absence of LPS (S1 Fig, panel C,D), suggestive of active production by moDCs in response to SEA. Stimulation of moDCs with LPS alone or LPS plus ω-1 did not drive accumulation of any of these compounds in the supernatants (S1 Fig, panel B). These results show that SEA contains a wide range of LMs as well as induces the release of particular LMs by DCs, including PGE_2_.

### Th2 polarization by SEA is dependent on PGE_2_ synthesis by moDCs in absence of ω-1

To test whether LMs present in SEA or generated by moDCs upon stimulation with SEA contribute to Th2 polarization by SEA, we stimulated moDCs with several of these LMs—in concentrations similar to those found in SEA or in supernatants of SEA-stimulated moDCs—and analyzed their ability to condition DCs to induce Th2 polarization. Amongst all tested lipids, we identified PGE_2_ as the only lipid capable of inducing Th2 polarization (Fig 1B). Based on this observation and given that ω-1 did not promote PGE_2_ synthesis by moDCs (Fig 1A), we wondered whether PGE_2_ may play a role in the previously observed ω-1–independent ability of SEA to prime Th2 responses [20]. To test this, we depleted ω-1 from SEA. Alongside ω-1, IPSE/α-1, which is another glycoprotein present in SEA but without Th2-priming capacity [20], was also depleted from this preparation. We found that treatment of moDCs with SEAΔα-1/ω-1 reduced expression of the Th1-polarizing cytokine interleukin 12 (IL-12) induced by LPS (Fig 1C) and promoted Th2 polarization as determined by increased IL-4, IL-5, and IL-13 secretion by T cells (Fig 1D). The Th2-polarizing effect of SEAΔα-1/ω-1 occurred both in the presence and absence of LPS (S2 Fig, panel A) but was less potent than Th2 polarization by total SEA (Fig 1D and S2 Fig, panel A). In contrast to complete SEA, we could hardly detect PGE_2_ in SEAΔα-1/ω-1 itself (time point 0 h in Fig 1E versus in Fig 1A), which suggests that during the depletion step of ω-1 and α-1 from SEA, PGE_2_ was removed from SEA as well. We observed that SEAΔα-1/ω-1 still promoted PGE_2_ synthesis in moDCs both with (Fig 1E) and without the presence of LPS (S1 Fig, panel D). This shows that SEA, in addition to containing PGE_2_ itself, stimulates PGE_2_ secretion by moDCs in an ω-1–independent fashion.

**Fig 2:**
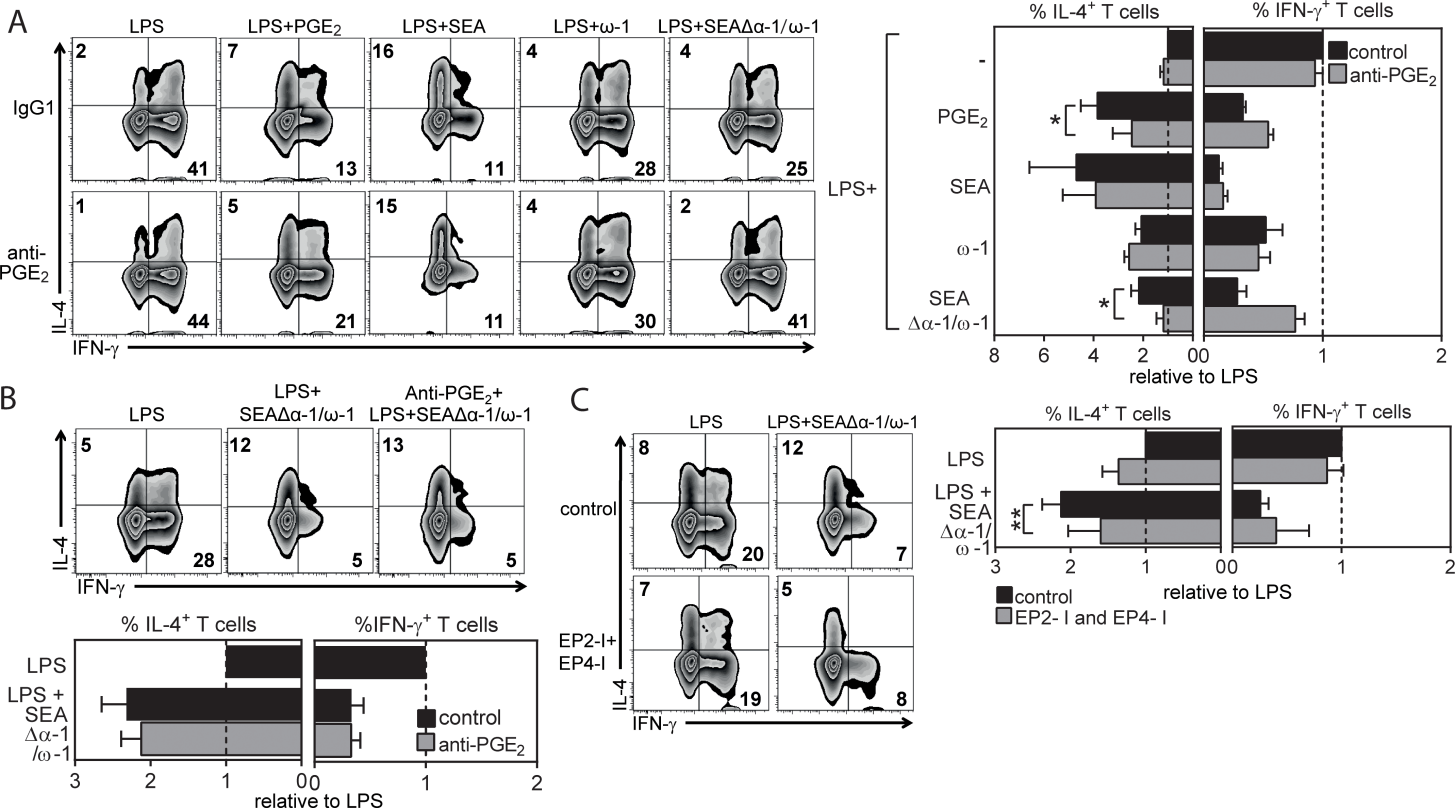
ω-1–independent Th2 polarization by SEA is dependent on PGE_2_ synthesis by moDCs. (A–C) T-cell polarization assay as described in Fig 1B. (A) Neutralizing anti-PGE_2_ antibody was added during stimulation of moDCs with indicated reagents or (B) during DC–T cell coculture. (C) EP2 and EP4 receptor inhibitors (EP2-I and EP4-I) were added during stimulation of moDCs with indicated stimuli. (A–C) Left: representative flow cytometry plots are shown of intracellular staining of CD4^+^ T cells for IL-4 and IFN-γ. Numbers in plots represent frequencies of cells in indicated quadrants. Right: these data were used to calculate the fold change in frequency of IL-4^+^ and IFN-γ^+^ T cells polarized by moDCs stimulated with indicated stimuli relative to the cytokine production by T cells polarized by LPS-stimulated moDCs, for which the values were set to 1. Bars represent mean ± SEM of at least 4 independent experiments. Significance was calculated based on the ratio of IL-4 over IFN-γ between conditions. *P < 0.05 and **P < 0.01 for significantly different from control conditions based on paired analysis (paired Student *t* test). Underlying data can be found in S1 Data. ω-1, omega-1; CD4, cluster of differentiation 4; EP2, prostaglandin E_2_ receptor 2; IL-4, interleukin 4; IFNγ, interferon γ; LPS, lipopolysaccharide; moDC, monocyte-derived DC; PGE_2_, prostaglandin E_2_; SEA, soluble egg antigen; Th2, T helper 2.

Next, we investigated the contribution of the synthesized PGE_2_ by SEA-stimulated moDCs to ω-1–independent Th2 induction. Strikingly, when PGE_2_ was neutralized using a specific anti-PGE_2_ antibody during stimulation of moDCs with SEAΔα-1/ω-1, the ability of SEAΔα-1/ω-1–stimulated moDCs to drive Th2 polarization was totally lost (Fig 2A). In contrast, neutralization of PGE_2_ in cultures of moDCs stimulated with ω-1 or complete SEA had no effect on the Th2-priming potential of these cells, which is consistent with our recently published study showing that ω-1—either alone or in the context of SEA—can prime Th2 responses via other mechanisms [9,10]. Moreover, we found that later neutralization of PGE_2_ limited to the coculture of SEAΔα-1/ω-1–stimulated moDCs with T cells did not impair Th2 polarization (Fig 2B), indicating that PGE_2_ synthesized by moDCs does not act as a polarizing signal on T cells but rather directly conditions moDCs in an autocrine manner to acquire a Th2-priming phenotype. In line with this observation, we found that simultaneous inhibition of the 2 major receptors of PGE_2_—prostaglandin E_2_ receptor 2 (EP2) and EP4—on moDCs reduced the ability of SEAΔα-1/ω-1–stimulated moDCs to induce a Th2 response (Fig 2C). These results collectively demonstrate that SEA, independently of ω-1, promotes PGE_2_ synthesis by moDCs, which subsequently, in an autocrine manner, conditions these cells to acquire a Th2-polarizing phenotype.

### OX40L is induced by SEA via PGE_2_ signaling and is required for Th2 induction

**Fig 3:**
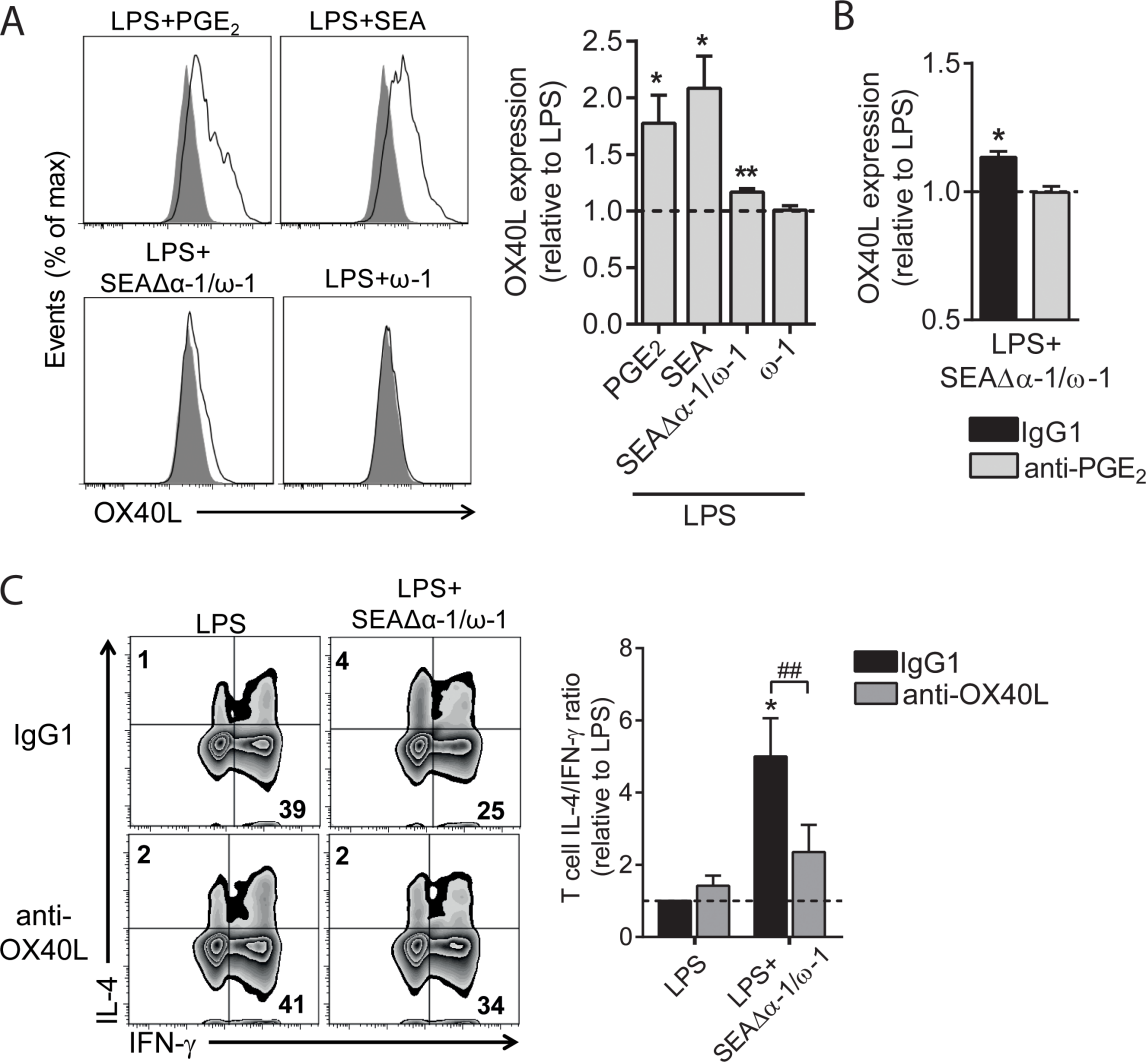
OX40L is induced by SEA via PGE_2_ signaling and is required for Th2 induction. (A, B) moDCs were stimulated as indicated for 48 h in the presence or absence of neutralizing anti-PGE_2_ antibody, after which expression of OX40L was analyzed by flow cytometry. The fold change based on geometric mean fluorescence is shown relative to LPS, which is set to 1 (dashed line). (A) PGE_2_ was taken along as positive control for OX40L expression and a representative histogram plot of OX40L expression is shown on the left. (C) T-cell polarization assay as described in Fig 2C. Neutralizing OX40L antibody was added during the DC–T cell coculture. Bar graphs represent means ± SEM of at least 6 independent experiments. “*”: *P* < 0.05: “**” and “##”: *P* < 0.01 for significant differences with the control conditions (*) or between-test condition (#) based on paired analysis (paired Student *t* test). Underlying data can be found in S1 Data. DC, dendritic cell; LPS, lipopolysaccharide; moDC, monocyte-derived DC; OX40L, OX40 ligand; PGE_2_, prostaglandin E2; SEA, soluble egg antigen; Th2, T helper 2.

moDCs matured in the presence of PGE_2_ are characterized by the expression of OX40L, a costimulatory molecule linked to Th2 polarization [26–28]. Moreover, an earlier study showed that moDCs stimulated with SEA express OX40L [3]. Indeed, we observed that stimulation of moDCs with PGE_2_, SEA, or SEAΔα-1/ω-1 induced expression of OX40L on moDCs either in the presence (Fig 3A) or absence of LPS (S2 Fig, panel B), whereas ω-1 did not induce OX40L expression (Fig 3A). While both SEAΔα-1/ω-1 and SEA promote PGE_2_ synthesis, OX40L induction by SEAΔα-1/ω-1 was lower than the levels induced by SEA. This might be explained by the fact that in contrast to SEAΔω-1/α-1, SEA additionally contains pre-existing PGE_2_ itself, resulting in higher overall concentrations of PGE_2_ that SEA-stimulated DCs are exposed to compared to SEAΔω-1/α-1–primed DCs. Neutralizing PGE_2_ prevented the induction of OX40L expression by SEAΔω-1/α-1 (Fig 3B and S2 Fig, panel C). Finally, neutralizing OX40L during the coculture with T cells significantly reduced the Th2-polarizing capacity of SEAΔα-1/ω-1–primed moDCs (Fig 3C and S2 Fig, panel D). These data demonstrate that OX40L expression by SEAΔα-1/ω-1–conditioned moDCs is dependent on PGE_2_ and that subsequently, induction of OX40L is important for Th2 polarization.

### SEA promotes PGE_2_ synthesis and drives Th2 polarization via Dectin-1 and Dectin-2 in human moDCs

**Fig 4:**
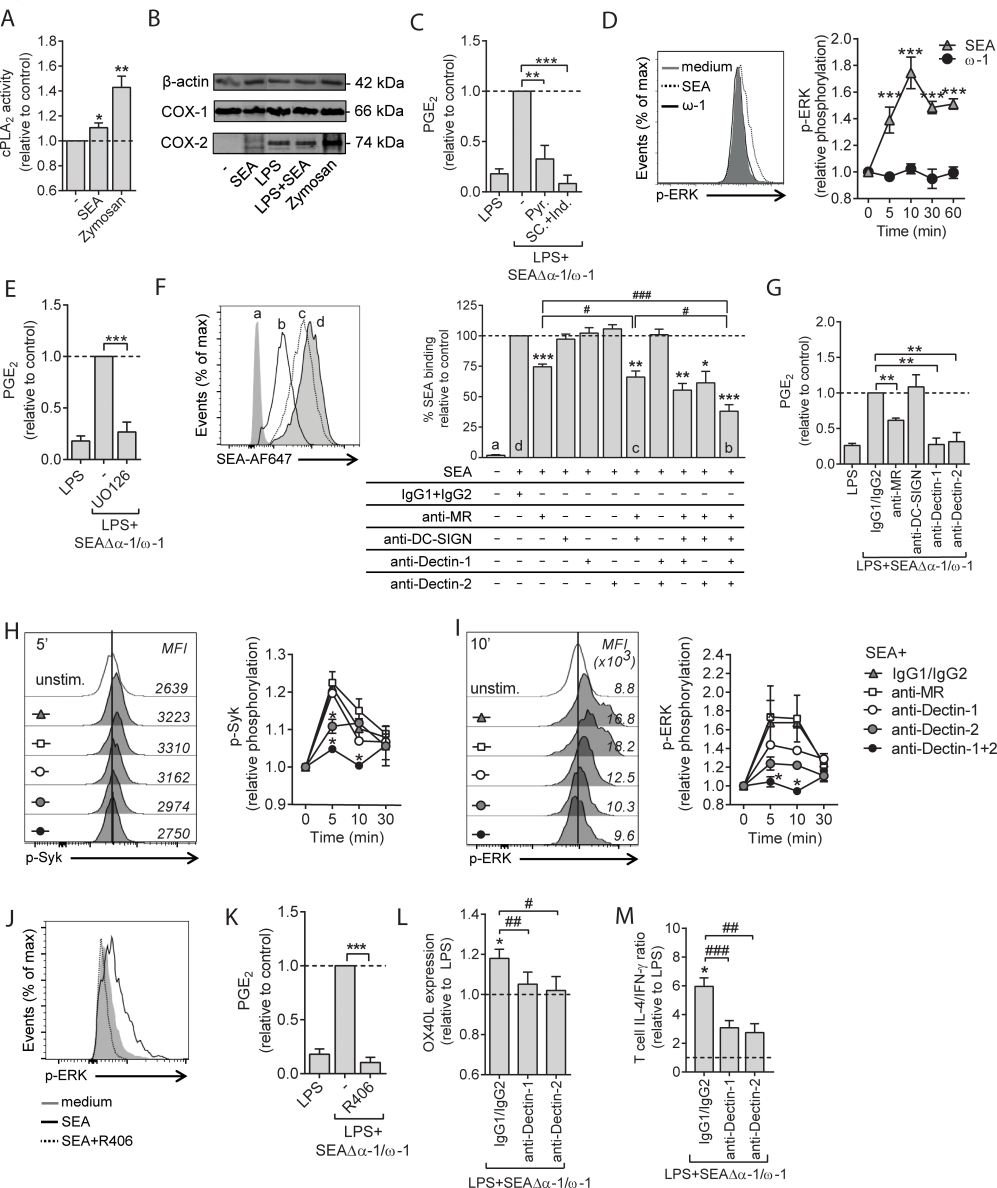
SEA promotes PGE_2_ synthesis and drives Th2 polarization via signaling through Dectin-1 and Dectin-2 in human moDCs. (A) cPLA_2_ activity 8 h after stimulation. Zymosan was taken along as a positive control for cPLA_2_ activation. (B) Protein expression of COX-1 and COX-2 were assessed by western blot. β-actin was used as housekeeping protein. One of 3 experiments is shown. (C) Following 1 h pre-incubation with specific inhibitors for cPLA_2_ (Pyr.) or COX-1 and COX-2 (SC and ind., respectively), moDCs were stimulated for 12 h with LPS plus SEAΔα-1/ω-1, and supernatants were collected for PGE_2_ determination by LC-MS/MS. (D) At the indicated time points after stimulation with depicted stimuli, phosphorylation of ERK was determined by flow cytometry. A representative flow cytometry plot of intracellular staining for phospho-ERK is shown on the left. (E) PGE_2_ levels were determined as in panel C. U0216 was used as inhibitor of ERK. (F) moDCs were treated 45 min with indicated blocking antibodies or isotype controls after which the cells were incubated with PF-647–labeled SEA. Antigen binding/uptake was analyzed by flow cytometry and plotted as relative differences. A representative flow cytometry plot of SEA uptake is depicted on the left. (G) PGE_2_ levels were assessed as in panel C, following pre-incubation with blocking antibodies as described in panel F. (H) Syk and (I, J) ERK phosphorylation were determined as described in panel D following pre-incubation with blocking antibodies as described in panel F or panel J with Syk inhibitor R406. Representative flow cytometry plots of Syk (panel H) and ERK (panel I, J) phosphorylation is shown on the left. (K) PGE_2_ levels were assessed as in panel C. (L, M) moDCs were pre-incubated with indicated blocking antibodies, followed by 48 h stimulation with LPS plus SEAΔα-1/ω-1, after which OX40L expression was determined by flow cytometry. Data are based on geometric mean florescence. (M) Cells described in panel L were used for T-cell polarization assay as described in Fig 2A. Data represent mean ± SEM of 2 (panel D, H, I) or at least 3 independent experiments (panel A, C–G, J–M) and are shown relative to control conditions, which are set to 1 (panel A, C–E, G–M) or 100% (F). “*” and “#”: *P* < 0.05; “**” and “##”: *P* < 0.01; “***” and “###”: *P* < 0.001 for significant differences with the control (*) or between-test condition (#) based on paired analysis (paired Student *t* test). Underlying data can be found in S1 Data. COX, cyclooxygenase; cPLA_2_, cytosolic phospholipase A_2_; DC-SIGN, dendritic cell-specific intercellular adhesion molecule-3-grabbing non-integrin; ERK, extracellular signal-regulated kinase; ind., Indometacin; LC-MS/MS, liquid chromatography tandem mass spectrometry; LPS, lipopolysaccharide; moDC, monocyte-derived DC; MR, mannose receptor; OX40L, OX40 ligand; PGE_2_, prostaglandin E_2_; Pyr., Pyrrophenone; SC, SC236; SEA, soluble egg antigen; Syk, spleen tyrosine kinase; Th2, T helper 2.

Classically, central to the synthesis of PGE_2_ is the release of AA from membrane phospholipids by cytosolic phospholipase A_2_ (cPLA_2_), which can then be converted into PGs, including PGE_2_, through constitutively expressed cyclooxygenase 1 (COX-1) and stimulus-induced COX-2. We observed that SEA induced a small but significant increase in cPLA_2_ activity (Fig 4A). We found that SEA did not change protein expression of COX-1, which was consistently expressed in all conditions. Moreover, SEA did not appear to promote COX-2 expression nor to alter LPS-driven COX-2 expression (Fig 4B), suggesting that SEA primarily promotes PGE_2_ synthesis through induction of cPLA_2_ activation. Indeed, selective inhibition of cPLA_2_ activity using pyrrophenone attenuated SEAΔα-1/ω-1–induced PGE_2_ synthesis (Fig 4C). For these experiments in which we analyzed the signaling events leading to PGE_2_ synthesis, we used PGE_2_-free SEAΔα-1/ω-1 and not PGE_2_-containing complete SEA in order to be able to selectively assess de novo synthesis of PGE_2_ by DCs. In addition, both COX-1 and COX-2 were important for PGE_2_ synthesis by SEAΔα-1/ω-1, as treatment of moDCs with COX-1 and COX-2 inhibitors indomethacin and SC236 abrogated SEAΔα-1/ω-1–driven PGE_2_ release (Fig 4C). IL-4 is known to suppress cPLA_2_ expression via IL-4Rα/γc signaling, while IL-13–driven signaling through IL-4Rα/IL-13Rα has been described to promote cPLA_2_ activity [29,30]. Because we cultured moDCs in the presence of IL-4, we wondered whether SEA might increase cPLA_2_ expression/activity by interfering with IL-4Rα/γc and/or enhancing IL-4Rα/IL-13Rα signaling to promote PGE_2_ synthesis. However, when we generated moDCs using IL-13 (S3 Fig, panel A)—which only signals through IL-4Rα/IL-13Rα—SEAΔα-1/ω-1 still promoted PGE_2_ production to similar levels as by IL-4–cultured moDCs (S3 Fig, panel B), suggesting that the SEA-induced increase in cPLA_2_ activity occurs through a different mechanism. Given that SEA has previously been reported to promote phosphorylation of extracellular-signal regulated kinase (ERK) [31] and that ERK can drive activation of cPLA_2_ [32], we evaluated the role of ERK in SEA-driven PGE_2_ synthesis. We found that SEA, in contrast to ω-1, induced phosphorylation of ERK (Fig 4D) and that inhibition of ERK signaling, using U0126, abrogated PGE_2_ synthesis induced by SEAΔα-1/ω-1 (Fig 4E).

Next, we aimed to identify the receptors through which SEA activates this pathway leading to PGE_2_ synthesis in moDCs. Previous studies have identified various c-type lectin receptors (CLRs) through which SEA can be recognized by antigen-presenting cells (APCs). For human moDCs primarily fucose- and mannose-binding dendritic cell-specific intercellular adhesion molecule-3-grabbing non-integrin (DC-SIGN) and the MR have been implicated in this process [13,33–35]. Moreover, studies with murine APCs have also pointed to a possible role in recognition of components in SEA for Dectin-1 and Dectin-2, which are classically known for their ability to bind and respond to β-glucans and α-mannans from fungal origins, respectively [36]. Yet whether components within SEA can be recognized and induce signaling via human Dectin-1 and/or Dectin-2 expressed by DCs remains to be determined. As a first step towards the identification through which receptor(s) SEA induces PGE_2_, we determined which of these receptors are involved in binding of SEA by moDCs. In line with earlier observations [9,13], blocking of the MR reduced binding of fluorescently labelled SEA, which could be further reduced when DC-SIGN binding was neutralized simultaneously. Blocking of both Dectin-1 and Dectin-2 in conjunction with MR plus DC-SIGN neutralization further reduced binding relative to blocking of just MR plus DC-SIGN, suggesting that all 4 receptors contribute to recognition of glycans or glycoproteins present in SEA (Fig 4F). Next, we aimed to identify through which of these CLRs SEA promotes PGE_2_ synthesis. We found that blocking of either Dectin-1 or Dectin-2, but not DC-SIGN, strongly attenuated PGE_2_ synthesis induced by SEAΔα-1/ω-1, while blocking the MR also resulted in reduced PGE_2_ synthesis albeit to a lesser extent. This suggests a major role for Dectin-1 and Dectin-2 in SEAΔα-1/ω-1–driven PGE_2_ synthesis (Fig 4G).

Dectins phosphorylate and activate spleen tyrosine kinase (Syk) through the immunoreceptor tyrosine-based activation motifs (ITAMs) present in their cytoplasmic domain (Dectin-1) or in the recruited Fc gamma receptor (FcRγ) chain (Dectin-2). Syk in turn can promote ERK phosphorylation [37]. Indeed, we observed that SEA stimulation resulted in phosphorylation of Syk, which was dependent on both Dectin-1 and Dectin-2 but not the MR (Fig 4H), and found that SEA-driven ERK phosphorylation was dependent on Dectin-1, Dectin-2, and Syk signaling (Fig 4I and 4J). Of note, blocking of either Dectin-1 or Dectin-2 alone only had minor effects on Syk and ERK phosphorylation, while these signals were totally blunted when both Dectin-1 and Dectin-2 signaling were blocked simultaneously (Fig 4H and 4I), suggesting that SEA depends on both receptors to activate this pathway in human moDCs. In line with these findings, inhibition of Syk signaling blunted SEAΔα-1/ω-1–induced PGE_2_ synthesis (Fig 4K). Finally, blocking either Dectin-1 or Dectin-2 attenuated OX40L expression (Fig 4L) as well as the Th2 response induced by SEAΔα-1/ω-1 (Fig 4M). Because signaling through Dectin-1 and Dectin-2 by fungal pathogen associated molecular patterns (PAMPs) is known to lead to IL-23 production and priming of Th17 responses [38,39], we analyzed IL-23 secretion and Th17 induction by SEA-primed DCs. Although we found that SEA promoted IL-23 release by DCs (S4 Fig, panel A), this did not result in any discernible Th17 polarization (S4 Fig, panel B). In conclusion, these data suggest that components in SEA promote PGE_2_ synthesis by moDCs through the MR, Dectin-1, and Dectin-2 and via a signaling cascade involving Syk, ERK, cPLA_2_, COX-1, and COX-2 that is required for ω-1–independent Th2 induction by SEA.

### PGE_2_ isomer generation via autoxidation contributes to Th2 induction by SEA

**Fig 5:**
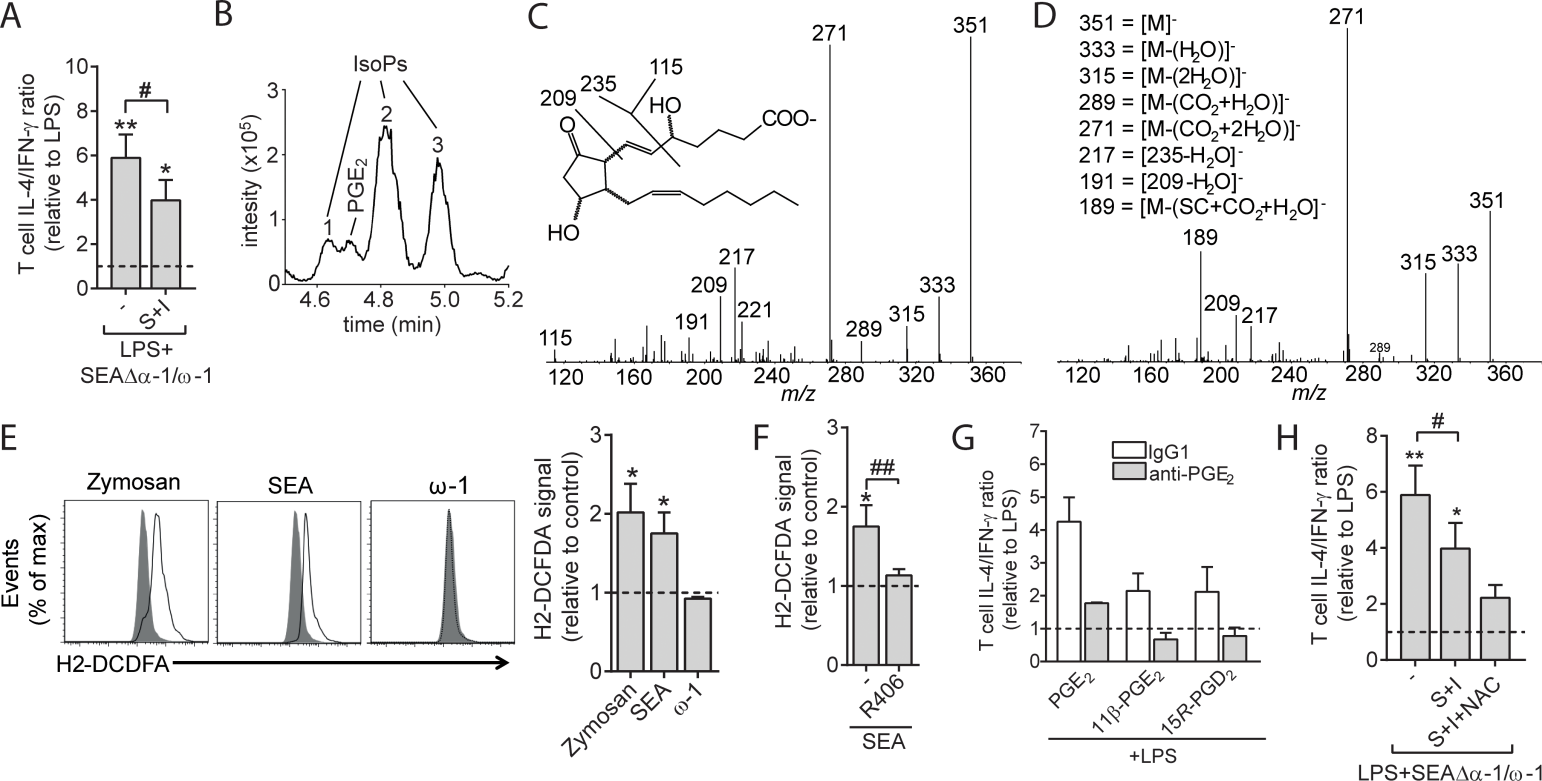
SEA-induced ROS production by moDCs results in PGE_2_ isomer synthesis that contributes to Th2 polarization. (A) T-cell polarization assay as described in Fig 2A in the presence of COX inhibitors S and I. Bars represent mean ± SEM of at least 3 independent experiments. (B) LC-MS/MS trace showing the transition *m/z* 351 → 271; the detected IsoPs are indicated by numbers. (C) Tandem MS spectrum of isomer 2, showing the fragment ion *m/z* 189, characteristic for 15-series IsoPs. (D) Showing the MS/MS spectrum of isomer 3, possibly identifying this isomer as a 5-series IsoP, based on the fragment ions *m/z* 115, 217, and 191. (E) ROS generation was determined by flow cytometry (H2-DCFDA) of moDCs pulsed for 6 h with indicated reagents. On the left, representative histograms for ROS induction are shown. On the right, the geometric mean fluorescence of these signals is enumerated and shown as fold change relative to unstimulated moDCs (dashed line set to 1). (F) ROS production was quantified as in panel E following pretreatment with general ROS scavenger NAC or R406 for 1 h. (G) moDCs were stimulated with indicated PGs with or without anti-PGE_2_ after which a T-cell polarization assay was performed as described in Fig 2A. (H) moDCs were stimulated with indicated reagents in the presence following 1 h pre-incubation with COX inhibitors (S and I) and NAC after which a T-cell polarization assay was performed as described in Fig 2A. Bars represent mean ± SEM of at least 3 independent experiments. “*” and “#”: *P* < 0.05: “**” and “#”: *P* < 0.01 for significant differences with the LPS control (*) or between-test conditions (#) based on paired analysis (paired Student *t* test). Underlying data can be found in S1 Data. COX, cyclooxygenase; H2-DCFDA, 2',7'-dichlorodihydrofluorescein diacetate; I, indomethacin; IsoP, isoprostane; LC-MS/MS, liquid chromatography tandem mass spectrometry; LPS, lipopolysaccharide; moDC, monocyte-derived DC; NAC, *N*-acetyl-*L*-cysteine; PGE_2_, prostaglandin E_2_; ROS, reactive oxygen species; S, SC236; SEA, soluble egg antigen; Th2, T helper 2.

The observations that PGE_2_ promoted Th2 induction by SEA and that this PGE_2_ synthesis was dependent on COX activity led us to hypothesize that blocking of COX activity in SEAΔα-1/ω-1–stimulated moDCs would abrogate their ability to prime Th2 responses. However, inhibition of COX-1 and COX-2 only partly reduced the Th2 response induced by these SEAΔα-1/ω-1–conditioned DCs (Fig 5A). A possible explanation for this unexpected result came from a careful reanalysis of the extracted ion chromatogram of the PGE_2_ trace (*m/z* 351 → 271) in which we noted that alongside PGE_2_, moDCs stimulated with SEA produced PGE_2_ isomers, also known as isoprostanes (IsoPs) (Fig 5B). Several studies have suggested that IsoPs may have similar properties as PGE_2_ but that, in contrast to the latter, they are generated by an autoxidation process directly from AA fueled by reactive oxygen species (ROS), independently of COX activity [40]. We found isomers “1” and “2” (Fig 5B) to have a fragment ion *m/*z 189 that is characteristic for 15-series IsoPs with identical relative retention times to commercially available 15*R*-PGD_2_ and 11β-PGE_2_, respectively, suggesting these isomers are 15*R*-PGD_2_ and 11β-PGE_2_ (Fig 5C) [41]. Isomer “3” showed a somewhat different tandem MS spectrum indicating it belongs to the class of 5-series IsoPs, but this could not be confirmed due to a lack of standard material (Fig 5D) [42].

To provide a mechanistic explanation for how SEA stimulation results in IsoP generation by moDCs, we test whether SEA could induce ROS production. Consistent with earlier observations in murine DCs [33,36], we observed that human moDCs stimulated with SEA and SEAΔα-1/ω-1, but not with ω-1, resulted in ROS production (Fig 5E) that was dependent on signaling through Syk (Fig 5F). To determine the biological significance of the generation of these two 15-series IsoPs in response to SEA, we first determined whether 15*R*-PGD_2_ and 11β-PGE_2_ IsoPs could affect T-cell polarization. These 2 IsoPs could condition moDCs for priming of a Th2 response, which could be blocked by treatment with anti-PGE_2_ (Fig 5G). In contrast to COX inhibition alone, pretreatment with COX inhibitors in conjunction with ROS scavenger *N*-acetyl-*L*-cysteine (NAC) abrogated the ability of SEAΔα-1/ω-1–stimulated moDCs to prime a Th2 response (Fig 5H), suggesting that enzymatically generated PGE_2_ and its isomers act in concert to condition moDCs for Th2 polarization.

### *S*. *mansoni* egg–driven Th2 polarization in vivo depends on Dectin-2 and Syk

**Fig 6:**
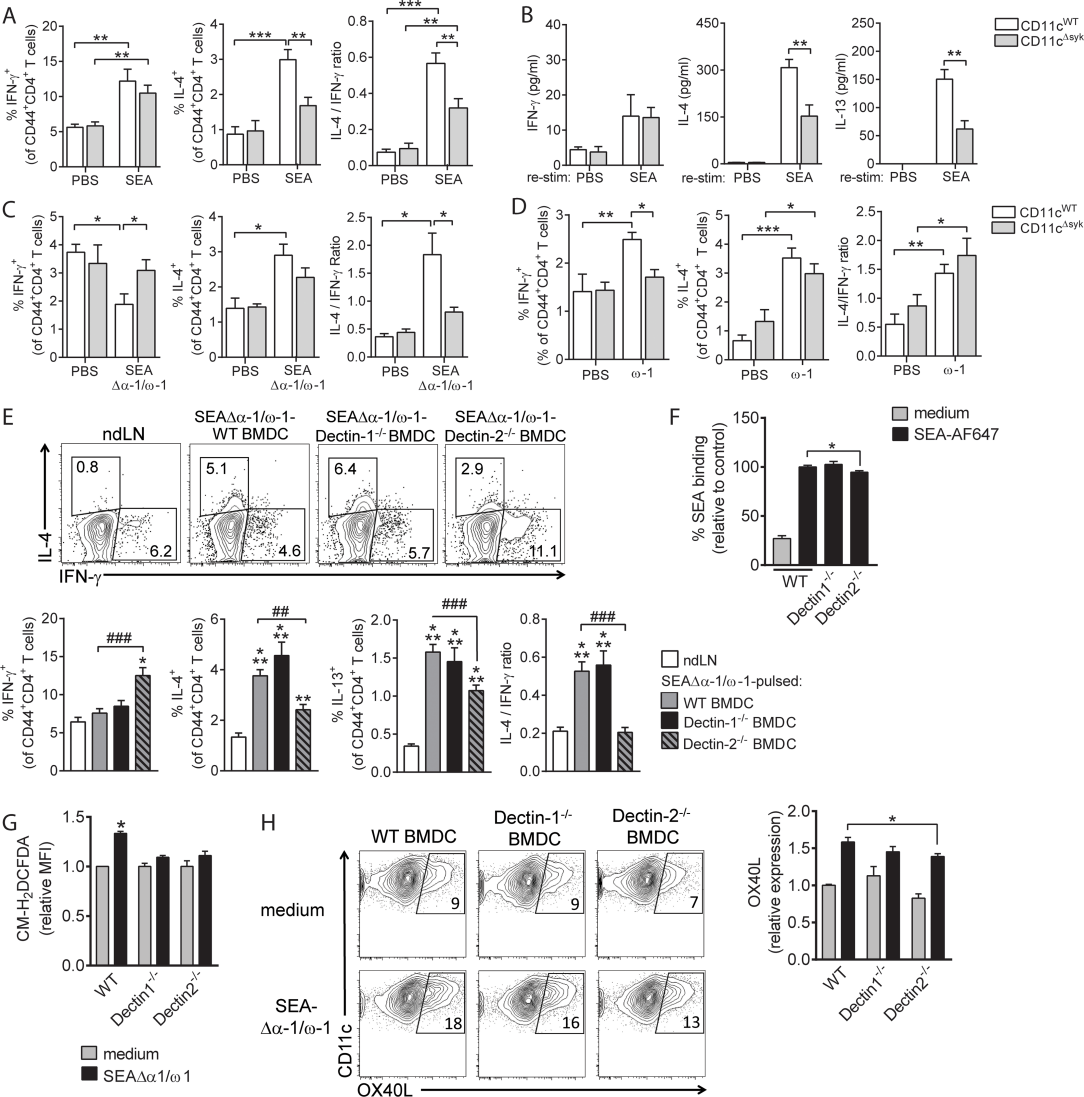
Th2 polarization induced by SEA is mediated via Dectin-2 and Syk signaling in vivo. (A–D) WT or CD11c^ΔSyk^ mice were injected with SEA (panel A, B), SEAΔα-1/ω-1 (panel C), or ω-1 (panel D) in the hind footpad, and draining pLNs were analyzed 7 d later. (A, C, D) pLN cells were restimulated with PMA/Ionomycin in the presence of brefeldin A, and CD4^+^ T cells were stained for indicated intracellular cytokines and percentage cytokine-positive CD4^+^ T cells enumerated. Based on these data, the ratio between the percentage IL-4^+^ over IFN-γ^+^ CD4^+^ T cells was determined as a measure for overall skewing towards Th2. (B) pLN cells were restimulated with SEA for 72 h, and cytokine levels in culture supernatants were determined. (A–D) Bar graphs represent mean ± SEM of 5 to 6 mice per group and are representative of 2 (panel A–C) or 1 (panel D) experiment. (E) BMDCs cultured from BM from WT, Dectin-1^−/−^, or Dectin-2^−/−^ mice were pulsed overnight with SEAΔα-1/ω-1 and injected into hind footpads after which CD4^+^ T-cell responses were analyzed as in panel A. Representative flow cytometry plots of intracellular staining of CD4^+^ T cells are depicted, of which the data are enumerated in bar graphs representing mean ± SEM of 2 independent experiments with 3 to 6 mice per group. (F) SEA binding and uptake was determined as in Fig 4F. (G) ROS production by indicated BMDCs was determined as described in Fig 5E, 1 h after stimulation with SEAΔα-1/ω-1. Based on MFI, bar graphs represent fold change relative to control condition, which is set to 1. (H) BMDCs were stimulated as indicated for 18 h after which expression of OX40L was analyzed by flow cytometry. Representative plots are depicted, of which the data are enumerated in bar graphs and shown as fold change relative to control condition, which is set to 1, based on percentage positive cells. (F–H) Bar graphs represent mean of duplicates or triplicates ± SEM of 2 independent experiments. “*”: *P* < 0.05; “**” and “##”: *P* < 0.01; “***” and “###”: *P* < 0.001 for significant differences with the control conditions (*) or between-test condition (#) based on unpaired analysis (unpaired Student *t* test). Underlying data can be found in S1 Data. ω-1, omega-1; BMDC, bone marrow–derived DC; CD4, cluster of differentiation 4; H2-DCFDA, 2',7'-dichlorodihydrofluorescein diacetate; IFN-γ, interferon γ; IL-4, interleukin 4; MFI, mean fluorescence intensity; nd/pLN, nondraining/popliteal lymph node; OX40L, OX40 ligand; PBS, phosphate buffered saline; PMA, phorbol 12-myristate 13-acetate; ROS, reactive oxygen species; SEA, soluble egg antigen; Syk, spleen tyrosine kinase; Th2, T helper 2; WT, wild-type.

We next aimed to assess the importance of this Dectin–Syk signaling in mediating Th2 polarization by *S*. *mansoni* egg antigens in vivo. First, to test the importance of Syk in Th2 polarization by DCs in response to egg antigen challenge in vivo, we made use of *Itgax*^cre^ *Syk*^fl/fl^ mice (CD11c^ΔSyk^), which selectively lack Syk expression in CD11c^+^ DCs. We found that, following subcutaneous immunization with SEA, cluster of differentiation 4 (CD4)^+^ T cells from draining lymph nodes (LNs) from CD11c^ΔSyk^ mice, compared to CD11c^WT^ controls, produced less Th2 cytokines ex vivo in response to both polyclonal (Fig 6A) and antigen-specific restimulation (Fig 6B), while interferon-γ (IFN-γ) production was not different between the 2 groups of mice. To explore the contribution of ω-1 in driving this Syk-dependent Th2 polarization by SEA in vivo, we additionally immunized these mice with SEAΔα-1/ω-1 and observed that CD11c^ΔSyk^ mice, also in response to this egg antigen mixture without ω-1, failed to significantly drive Th2 polarization (Fig 6C). Skewing towards Th2 following immunization with ω-1, however, was unaffected by loss of Syk in the DCs (Fig 6D), showing that ω-1 drives a Th2 response through a Syk-independent route as well as ruling out a role for ω-1 in driving Syk-dependent Th2 polarization by SEA in vivo. This is in line with our in vitro findings with human moDCs and provides evidence that Syk signaling in DCs plays a key role in Th2 priming by *S*. *mansoni* egg antigens in vivo.

Next, to establish the role of Dectin-1 and/or Dectin-2 in driving this Syk-dependent response in vivo, we assessed the ability of SEAΔα-1/ω-1–pulsed bone marrow–derived DCs (BMDCs) derived from Dectin-1–deficient (*Clec7a*^*−/−*^) and Dectin-2–deficient (*Clec4n*^−/−^) mice to prime Th2 responses in vivo following adoptive transfer into wild-type (WT) recipient mice. We found that SEAΔα-1/ω-1–pulsed Dectin-2^−/−^ BMDCs—just like SEAΔα-1/ω-1–pulsed CD11c^ΔSyk^ BMDCs—failed to prime a Th2 response, whereas Th2 induction by Dectin-1^−/−^ BMDCs was not significantly affected (Fig 6E and S5 Fig, panel A,B). Furthermore, Th17 responses were suppressed by SEAΔα-1/ω-1–pulsed WT BMDCs, which was even further reduced in draining LNs of mice immunized with SEAΔα-1/ω-1–pulsed Dectin-1^−/−^ BMDCs (S6 Fig). Finally, in accordance with the impaired ability of Dectin-2^−/−^ and CD11c^ΔSyk^ BMDCs to prime Th2 responses, these cells—but not Dectin-1^−/−^ BMDCs—displayed a reduced uptake of SEA (Fig 6F), ROS production (Fig 6G), and OX40L expression in response to SEAΔα-1/ω-1 stimulation (Fig 6H and S5 Fig, panel C-E). Together, these data reveal that Dectin-2 and Syk signaling in DCs play a key role in Th2 priming by *S*. *mansoni* egg antigens in vivo independently of ω-1.

**Fig 7:**
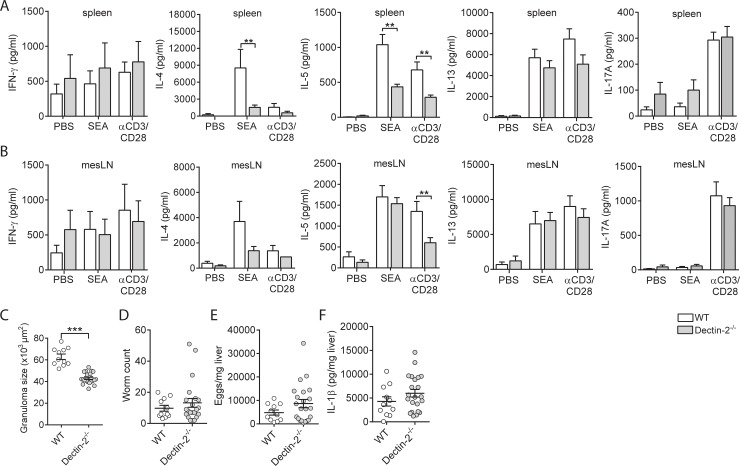
Dectin-2 signaling is required for induction of a Th2 response during *S*. *mansoni* infection. WT and Dectin-2^−/−^ mice were infected with *S*. *mansoni*. After 8 wk of infection, cells from spleens (A) or mLNs (B) were restimulated with SEA or anti-CD3/CD28 for 72 h, and cytokine levels were analyzed in supernatants by Luminex or ELISA. Bars represent mean ± SEM of combined data of at least 2 or 3 independent experiments with 5 to 10 mice per group. (C) Granuloma sizes around eggs trapped in the liver of 8-wk–infected mice were assessed in Masson blue–stained liver sections. Data are based on 10 mice per group. Number of worms (D) and liver and intestinal eggs (E) in mice infected with *S*. *mansoni* for 8 wk. (F) IL-1β protein levels in livers of mice infected with *S*. *mansoni* for 8 wk. **P < 0.01 and ***P < 0.001 for significant differences relative to the control mice based on unpaired analysis (unpaired Student *t* test). Underlying data can be found in S1 Data. CD3, cluster of differentiation 3; IL-1β, interleukin 1β; mLN, mesenteric lymph node; SEA, soluble egg antigen; Th2, T helper 2; WT, wild-type.

Finally, we set out to assess the importance of this signaling axis in Th2 differentiation and Th2-driven immunopathology during a natural infection with *S*. *mansoni*, using Dectin-1^−/−^ and Dectin-2^−/−^ mice. During *S*. *mansoni* infection, adult worms residing in the portal vasculature release eggs that get trapped in the liver, where they induce strong Th2 responses that orchestrate the development of granulomatous lesions surrounding the eggs [8]. The intensity of the Th2 response and associated granulomatous inflammation peaks at 8 wk after infection. To compare the Th2 response induced by this infection in WT and Dectin-1^−/−^ and Dectin-2^−/−^ mice, cells from mesenteric LNs and spleens from 8-wk–infected mice were restimulated with SEA or anti-CD3/CD28. We found that infected Dectin-2^−/−^ mice displayed lower production of Th2 cytokines IL-4 and IL-5 in both lymphoid organs in comparison to their infected WT counterparts, while IFN-γ production was not different between the 2 groups (Fig 7A and 7B). In infected Dectin-1^−/−^ mice, only IL-5 production by splenocytes was reduced (S7 Fig, panel A,B). Of note, no SEA-specific Th17 responses, as determined by IL-17A production, were detected in spleen and mesenteric LNs (mLNs) of infected mice (Fig 7A and 7B and S7 Fig, panel A,B). In line with the reduced Th2 responses found in the infected Dectin-2^−/−^ mice, granuloma size around the eggs trapped in the liver was smaller relative to infected WT mice (Fig 7C). This difference in Th2 response was not due to differences in infection load because both Dectin-2^−/−^ and WT mice were found to harbor similar numbers of eggs and adult worms (Fig 7D and 7E). Dectin-1^−/−^ mice did not show an altered granulomatous response towards liver eggs (S7 Fig, panel C), nor did they show differences in numbers of adult worms and eggs (S7 Fig, panel D,E). We previously found that inflammasome activation, which can be triggered by SEA via Dectin-2 signaling, can alter T-cell polarization and contribute to granuloma formation during *S*. *mansoni* infection [33]. However, levels in IL-1β protein, as readout for inflammasome activity, in liver were similar between Dectin-2^−/−^ and WT mice (Fig 7F). Altogether, these findings highlight an important role for Dectin-2 in promoting Th2 differentiation and immunopathological outcome of this response during *S*. *mansoni* infection.

## Discussion

The molecular mechanisms through which DCs prime Th2 responses, including those elicited by helminths, are still incompletely understood. We here explored the role of PUFAs and LMs in Th2 induction by *S*. *mansoni eggs*, which are well known for their potent ability to elicit strong Th2 responses. This enabled us to identify a novel signaling axis in DCs involving Dectin-Syk-PGE_2_-OX40L through which Th2 responses are induced in vitro and in vivo.

Some studies have documented that different life stages of *S*. *mansoni* are able to produce LMs from both COX products (e.g., PGE_1_, PGE_2_, PGD_2_, and PGA_2_) and Lipoxygenase products (e.g., Leukotriene B_4_, 5-Hydroxyeicosatetraenoic acid [5-HETE], 12-HETE) [40,43]. However, the existence of LMs in eggs or SEA had not been examined before. Here, we uncovered that SEA itself contains various PUFAs and LMs with potential immunomodulatory properties. In particular, the presence of the well-studied immunomodulatory eicosanoid PGE_2_ caught our attention because, among the pleiotropic properties that have been attributed to this lipid, it has been associated with promoting Th2 polarization by functional modulation of DCs [3,27]. Moreover, we found PGE_2_ not only to be present in SEA but also to be synthesized by DCs themselves in response to SEA stimulation. While other life cycle stages of *S*. *mansoni* have been shown to promote PGE_2_ synthesis in host cells such as cercariae in keratinocytes [44], we are the first to report and mechanistically investigate the ability of egg-derived antigens to promote PGE_2_ synthesis in immune cells. We found that this PG, in contrast to several other LMs secreted by SEA-stimulated DCs, was not only sufficient to condition moDCs for Th2 polarization but also crucial for mediating ω-1–independent Th2 polarization by SEA. This identifies PGE_2_ as a key factor through which SEA, independently of ω-1, primes Th2 responses. A question that remains to be answered is what the relative contribution is of the pre-existing PGE_2_ (present in SEA) versus PGE_2_ synthesized by moDCs in mediating the Th2-polarizing effect. The observation that SEA from which ω-1 was depleted was fully dependent on de novo–synthesized PGE_2_ by the moDCs for Th2 polarization at least shows that moDC-derived PGE_2_ can be sufficient for promoting a Th2 response. Moreover, the fact that SEA requires Syk signaling in DCs to prime a Th2 response in vivo would argue that PGE_2_ derived from SEA itself is insufficient to condition DCs for Th2 priming and that SEA instead rather depends on Syk-driven de novo PGE_2_ synthesis for this response.

Mechanistically, we found that PGE_2_ derived from moDCs acts in an autocrine manner to promote Th2 polarization by promoting the expression of OX40L in moDCs. OX40L expression has been shown to be important for Th2 polarization by DCs stimulated with various other Th2-priming stimuli, such as allergens and thymic stromal lymphopoietin (TSLP) [28,45,46]. Congruent with these studies, we found that OX40L expression was crucial for Th2 polarization by SEA from which ω-1 was depleted. While expression of OX40L in response to SEA has been documented before [3,12], we now provide evidence that SEA-induced OX40L expression in moDCs is secondary to its ability to induce PGE_2_ synthesis by these cells.

Moreover, we found that SEA from which ω-1 was depleted was dependent on signaling through both Dectin-1 and Dectin-2 to condition moDCs to drive Th2 polarization. Dectin-2 has been linked to Th2 polarization before in the context of allergic responses induced by house dust mite [47,48]. In these studies, Dectin-2 was found to mediate Th2 induction through the generation of cysteinyl leukotrienes by murine DCs. However, we did not observe induction of cysteinyl leukotrienes by SEA. Instead, we found that SEA interacts with Dectin-1, Dectin-2, and the MR to promote PGE_2_ synthesis. Downstream of these receptors, we identified a pathway involving, Syk, ERK, and cPLA_2,_ that leads to the release of AA, which subsequently acts as a substrate for COX to produce PGE_2_. We currently do not have a clear explanation for the observations that PGE_2_ synthesis and Th2 induction by human moDCs can be blocked by either anti–Dectin-1 or Dectin-2 antibody while full inhibition of Syk phosphorylation is only seen when both receptors are blocked. Possibly, this suggests that Syk phosphorylation needs to reach a certain threshold level before it can start relaying signals leading to PGE_2_ synthesis and Th2 polarization. In this scenario, reducing Syk phosphorylation below that threshold (seen with single CLR blockade) would be sufficient to block PGE_2_ synthesis/Th2 induction, and further inhibition of Syk phosphorylation would not have additional functional consequences.

The observation that the MR also seems to play a role in SEA-driven PGE_2_ synthesis—despite the fact that the MR itself, in contrast to Dectin-1 and Dectin-2, does not harbor an intracellular signaling motif—leads us to speculate that the MR may collaborate with Dectin-1 and/or Dectin-2 to form heterodimers or multimers that effectively bind glycans or glycoproteins in SEA that allow for efficient activation of the signaling cascade downstream of Dectins resulting in PGE_2_ synthesis. Associations of different CLRs to potentiate glycan-induced signaling have been described before for Dectin-2 and Dectin-3 [49]. Glycans derived from the cell wall of fungi such as *Candida albicans* [50] are well known to promote PGE_2_ synthesis through this pathway via activation of Dectin-1, Dectin-2, and the MR [39,51]. However, in this context, the production of PGE_2_ seems to contribute to Th17 priming by APCs and not Th2 [39]. This difference might be explained by differences in glycan repertoire between fungi and schistosome eggs. For instance, classical β-glucans expressed by fungi are not present in SEA [52]. Therefore, the carbohydrates in SEA that mediate Dectin binding may interact differently with, or have a lower affinity for, these receptors than fungal carbohydrates do. This may induce a qualitatively and/or quantitatively different signaling cascade that could trigger sufficient PGE_2_ synthesis and OX40L expression to allow for Th2 induction to occur, without promoting the expression of high levels of cytokines required for Th17 polarization. Secondly, fungal Dectin agonists may trigger additional pattern-recognition receptors (PRRs) that are not activated by SEA to induce pro-inflammatory cytokine expression [53]. Finally, the immunological outcome of Dectin engagement can also be cell type dependent. For instance, Dectin-1 ligand curdlan was found to promote Th2 responses via plasmacytoid DCs, while this same ligand conditioned myeloid DCs to inhibit Th2 responses [54] or to promote Th9 responses [55]. Similar to our observations with SEA, the conditioning of plasmacytoid DCs by curdlan to promote Th2 responses was dependent on induction of OX40L expression [54]. Currently, studies are underway to identify the glycoproteins or glycan moieties present in SEA that bind to Dectin-1, Dectin-2, and the MR to promote this response and identify how these receptors collaborate to promote Syk activation and PGE_2_ synthesis.

We provide evidence that COX-independent generation of several PGE_2_ isomers (IsoPs) by SEA, independently of enzymatically synthesized PGE_2_, is capable of conditioning moDCs for Th2 priming. While COX-independent generation of these isomers has been described before, as a result of auto-oxidation of AA by free radicals [40], we now here show a role for these IsoPs in regulation of an immune response. In line with the free radical–dependent synthesis of IsoPs, we found that SEA could drive ROS production in a Syk-dependent manner, which corroborates a recent study showing that SEA can induce ROS in murine DCs [33]. However, the latter study focused on the role of ROS in SEA-driven inflammasome activation and did not report on other ROS-mediated effects. Our data suggest that enzymatically synthesized PGE_2_ and its ROS-induced isomers act in concert in Dectin-mediated conditioning of moDCs for Th2 priming by SEA. Moreover, we found that the widely used neutralizing anti-PGE_2_ antibody [56] that we have used in this study not only neutralizes PGE_2_ but also harbors cross-reactivity towards 2 of the main IsoPs that we found to be generated by SEA-stimulated DCs. This can explain our observation that PGE_2_ neutralization, in contrast to COX inhibition, did fully block Th2 polarization.

Our findings that murine DCs that are deficient for Dectin-2 or Syk display reduced OX40L expression following exposure to SEA and fail to mount a Th2 response in vivo provide strong support for a key role of the Dectin-Syk-PGE_2_-OX40L axis in Th2 polarization by *Schistosoma* egg–derived antigens in vivo. Our studies with *S*. *mansoni*–infected Dectin-2^−/−^ mice suggest that also during natural infection, this signaling axis seems to be crucial for induction of Th2 responses. Nonetheless, our in vivo work can currently not formally exclude the possibility that apart from PGE_2_, there are other factors downstream of Dectin–Syk signaling that contribute to Th2 priming by *S*. *mansoni*. For instance, SEA has previously been reported to activate the Nlrp3-inflammasome in a Dectin-2–dependent manner, and inflammasome-deficient mice were shown to have an altered T-cell polarization profile and a reduction in granuloma size during *S*. *mansoni* infection similar to our observations reported here in infected Dectin-2^−/−^ mice. However, the fact that—in contrast to what was observed in inflammasome-deficient mice [33]—there was no reduction in total IL-1β levels in livers of Dectin-2^−/−^ mice during *S*. *mansoni* infection would suggest that, downstream of Dectin-2, the PGE_2_-OX40L axis rather than inflammasome activation plays a key role in Th2 priming during, and in the immunopathological outcome of, this infection. Nonetheless, additional studies will be needed to definitively determine the individual contribution of each pathway to the immunopathology in vivo. Somewhat surprising was the observation that in contrast to the in vitro data with human moDCs, Dectin-1 appears to be less important for Th2 polarization in vivo in mice. This may suggest that murine Dectin-1, as opposed to its human counterpart, does not play a major role in recognition of glycans present in SEA, which, although currently still speculation, might be due to differences in glycan specificity or in expression of Dectin-1 isoforms between murine and human DCs [38,57]. More detailed comparative studies between the SEA-binding characteristics of human and murine Dectin-1 could provide more molecular insight in the mechanisms underlying the difference in requirement for Dectin-1 in Th2 polarization by SEA between the human in vitro and murine in vivo models.

**Fig 8:**
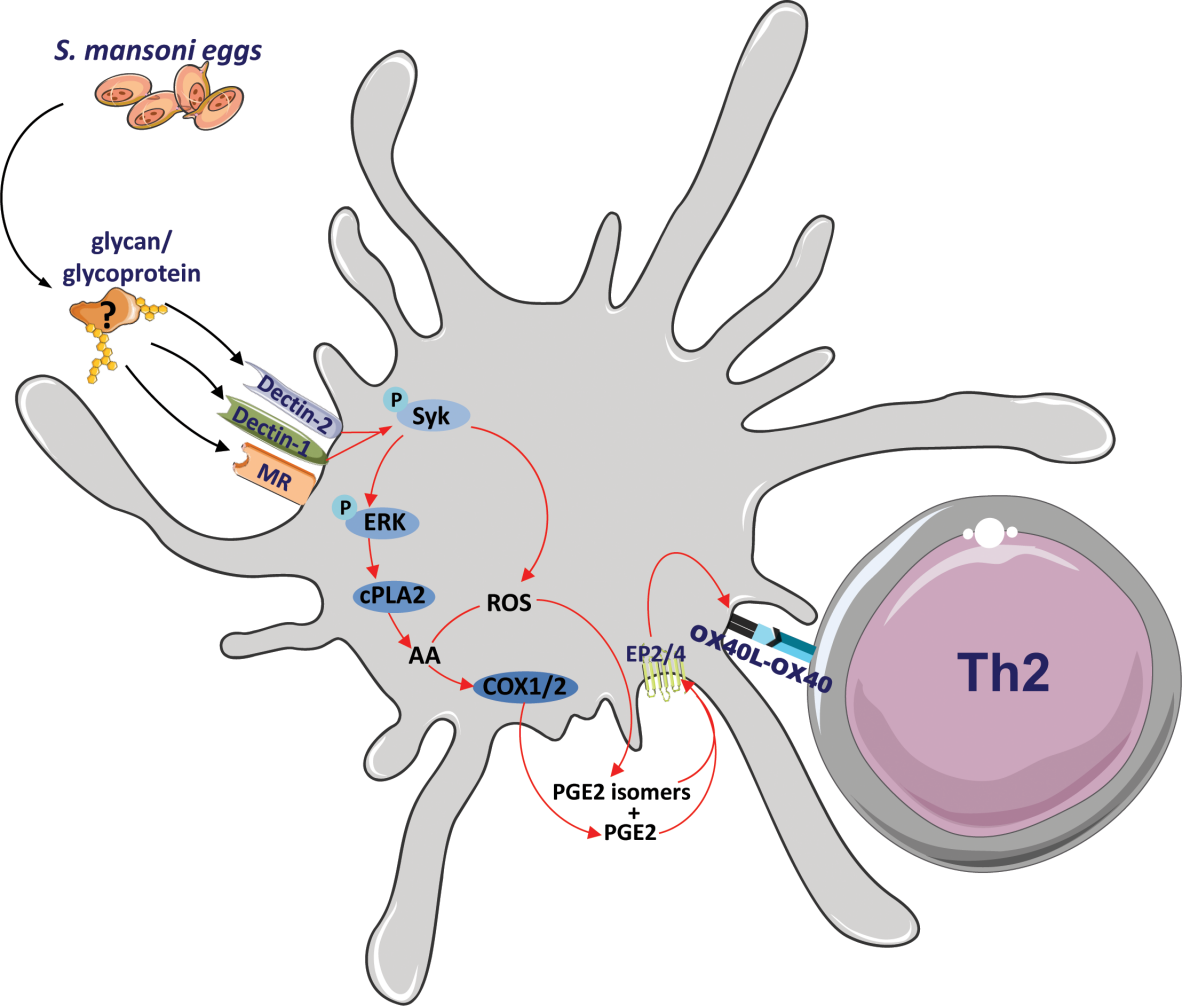
Proposed model of *S*. *mansoni*–driven Th2 polarization. *S*. *mansoni* egg antigens, but not ω-1, interact with Dectin-1 and Dectin-2 expressed by DCs to promote 2 intracellular pathways in moDCs in an Syk-dependent manner: ERK-cPLA_2_-COX and ROS activity that culminate in PGE_2_ and PGE_2_ isomer synthesis, respectively. Both PGE_2_ and its isomers can then bind to EP2 and EP4 in an autocrine loop to trigger OX40L expression, which endows the DCs with the capacity to prime a Th2 response. ω-1, omega-1; COX, cyclooxygenase; cPLA_2_, cytosolic phospholipase A_2_; DC, dendritic cell; EP2, prostaglandin E_2_ receptor 2; ERK, extracellular signal-regulated kinase; moDC, monocyte-derived DC; OX40L, OX40 ligand; PGE_2_, prostaglandin E_2_; ROS, reactive oxygen species; Syk, spleen tyrosine kinase; Th2, T helper 2.

In summary, we propose a model (Fig 8) in which SEA can condition DCs for Th2 polarization independently of ω-1 by triggering Dectin-1, Dectin-2, and the MR to induce in a Syk-dependent fashion the synthesis of PGE_2_ and IsoPs, which subsequently promote OX40L expression in an autocrine manner. OX40L then enables the SEA-stimulated moDCs to prime a Th2 response. The fact that neutralization of PGE_2_ and its isomers completely blunted the Th2-priming ability of moDCs that had been stimulated with SEA from which ω-1 had been depleted provides strong support for the notion that this pathway can fully account for “residual” ability of SEA to prime Th2 polarization in the absence of ω-1. However, the fact that this same intervention had little or no effect on the Th2-priming capacity of complete SEA in our in vitro moDC assay suggests that the Dectin-PGE_2_-OX40L signaling axis can be compensated for by the presence of ω-1, which employs distinct mechanisms to condition moDCs for Th2 polarization, and that this novel axis is only unmasked in vitro when ω-1 is removed from SEA. In vivo, however, the contribution of Dectin-PGE_2_-OX40L signaling axis in egg antigen-driven Th2 polarization appears to be much more dominant, given that interference with Syk or Dectin signaling did result in an impaired Th2 response induced by complete SEA or by a natural infection with *S*. *mansoni*, respectively. This would be corroborated by our previous work showing that SEA from which ω-1 was depleted was still as potent in inducing a Th2 response as complete SEA in vivo [20] and that ω-1 knockdown in *S*. *mansoni* eggs by lentiviral transduction did not reduce Th2 responses induced by the eggs in vivo [19]. Together, this leads us to speculate that in vivo, the Dectin-PGE_2_-OX40L signaling axis plays a more important role in schistosome egg-driven Th2 polarization than the one that is induced by ω-1 in which the MR and suppression of protein synthesis play a central role [9].

In conclusion, we have delineated a previously unrecognized pathway involving Dectin-1/Dectin-2, PGE_2_, and OX40L through which Th2 immunity is induced. It is interesting to note that antigens from liver fluke *Fasciola hepatica* as well as from house dust mite have been shown to be recognized by Dectin-1 [58] and Dectin-2 [59], respectively, which makes it tantalizing to speculate that this Dectin-PGE_2_–dependent signaling axis in DCs is not only triggered by *S*. *mansoni* but possibly also by other helminths or allergens to promote Th2 responses. As such, targeting this axis may hold promise as an approach to regulate type 2 immune responses for therapeutic purposes, not only in the context of schistosomiasis but possibly also in other helminth infections and in major diseases of the Western world, such as allergies and type 2 diabetes, that are caused by overzealous and defective type 2 immune responses, respectively.

## Materials and methods

### Ethics statement

All animal experiments were performed in accordance with local government regulations and the EU Directive 2010/63EU and Recommendation 2007/526/EC regarding the protection of animals used for experimental and other scientific purposes as well as approved by the Regierung von Oberbayern (animal license number 55.2-1-54-2532-28-1), the animal ethics committee at CNIC (animal license number CNIC10-16-PROEX 240/16), and the Dutch Central Authority for Scientific Procedures on Animals (CCD) (animal license number AVD116002015253). For infection and immunization experiments as described in this study, animals were euthanized using cervical dislocation or an overdose of ketamine/xylazine.

### Mice

*Clec7a*^−/−^ and *Clec4n*^−/−^ mice (C57BL/6) were housed and bred at the MIH, TUM, Germany, under SPF conditions. *Itgax*^cre^ *Syk*^fl/fl^ mice [60] were housed and bred at the CNIC, Madrid, Spain, under SPF conditions.

### Preparation and purification of *S*. *mansoni* egg–derived antigens

SEA, IPSE/α-1, ω-1, and SEAΔα-1/ω-1 from *S*. *mansoni* eggs were prepared and isolated as described previously [3,20].

### Human DC culture, stimulation, and analysis

Peripheral blood mononuclear cells were isolated from venous blood of healthy volunteers by density centrifugation of Ficoll as described before [10]. Monocytes were isolated by positive magnetic cell sorting using CD14-microbeads (Miltenyi Biotech, Bergisch Gladbach, Germany) and cultured in 10% FCS RPMI medium supplemented with 20 ng/mL rGM-CSF (BioSource/Invitrogen, Carlsbad, CA) and 0.86 ng/mL of rIL-4 (R&D Systems, Minneapolis, MN). On day 2, medium including supplements was replaced. In the presence or absence (if indicated) of 100 ng/mL ultrapure LPS (*Escherichia coli* 0111 B4 strain; InvivoGen, San Diego, CA), immature moDCs were stimulated on d 6 with indicated reagents: SEA (50 μg/mL), ω-1 (500 ng/mL), IPSE (500 ng/mL), SEAΔα-1/ω-1 (50 μg/mL), PGE_2_ (2.5 ng/mL; Cayman Chemical, Ann Arbor, MI), IFN-γ (1,000 U/mL) as Th1 control, or 0.1 mg/mL Zymosan (Z4250; Sigma-Aldrich, St. Louis, MO). Alternatively, moDCs were stimulated with 2.5 ng/mL LXA_4_, 2.5 ng/mL PGD_2_, 12.5 μg/mL 5-HETE, 12.5 μg/mL 8-HETE, 12.5 μg/mL 11-HETE, 25 μg/mL 9-HODE, or 25 μg/mL 13-HODE (all Cayman Chemical). For blocking experiments, cells were pre-incubated for 60 min at 37°C with 20 μg/mL anti–DC-SIGN (clone AZN-D1; Beckman-Coulter, Fullerton, CA), anti-MR (clone 15.2; Biolegend, San Diego, CA), anti–Dectin-1 (clone #259931; R&D Systems), anti–Dectin-2 (clone Q7-4B5, InvivoGen), 20 μg/mL IgG1 control antibody for both anti–DC-SIGN and anti-MR, IgG2a (clone RTK2758; Biolegend) control antibody for anti–Dectin-1 and anti–Dectin-2, 1 μM R406, 4 μM UO126 (Merck, Amsterdam, the Netherlands), 1 μM Pyrrophenone (Merck), 10 μM SC-236 (Sigma-Aldrich) in combination with 10 μM Indometachine (Sigma-Aldrich), 10 μg/mL neutralizing anti-PGE_2_ antibody (2B5; Cayman Chemical), 10 μg/mL IgG1 antibody (clone MOPC-21; Biolegend) as control for anti-PGE_2_ antibody, 10 μM EP2 (AH6809; Cayman Chemical), or 10 μM EP4 (AH23848; Cayman Chemical) receptor antagonist. After 24 or 48 h of stimulation, surface expression of costimulatory molecules was determined by flow cytometry (FACS-Canto; BD Biosciences, Breda, the Netherlands) using the following antibodies: CD14 (clone MΦP9), CD86 (clone 2331 FUN-1), CD40 (clone 5C3), and CD80 (clone L307.4) (all BD Biosciences); HLA-DR (clone LN3) (eBioscience, San Diego, CA); CD83 (clone HB15e) and CD1a (clone BL6) (both Beckman-Coulter); and CD252/OX40L (clone ANC10G1; Ancell, Bayport, MN). Only live cells that were negative for 7-AAD (eBioscience) were included in the analysis. Routinely, in all tested culture conditions, at least 85% of the cells were alive.

### Cytokine detection

A total of 1 × 10^4^ moDCs matured for 48 h were cocultured with 1 × 10^4^ CD40L-expressing J558 cells for 24 h, and supernatants were collected to determine IL-12p70 levels, using mouse anti-human IL-12 (Clone 20C2) as capture antibody and biotinylated mouse anti-human IL-12 (Clone C8.6) (both BD Biosciences) in a sandwich ELISA. IL-23 levels were quantified in supernatants of moDCs stimulated for 40 h by ELISA.

### Human DC and T cell coculture and determination of T-cell polarization

For analysis of T-cell polarization, 5 × 10^3^ moDCs pulsed for 48 h were cultured with 2 × 10^4^ allogenic naive CD4^+^ T cells for 11 d in the presence of *Staphylococcal enterotoxin B* (10 pg/mL). On d 6 and 8, rhuIL-2 (10 U/mL; R&D Systems) was added to expand the T cells. Intracellular cytokine production was analyzed after restimulation with 100 ng/mL phorbol myristate acetate plus 2 μg/mL ionomycin for 6 h; 10 μg/mL brefeldin A was added during the last 4 h. Subsequently, the cells were fixed with 1.9% paraformaldehyde (all Sigma-Aldrich). The cells were permeabilized with 0.5% saponin (Sigma-Aldrich) and stained with antibodies against IL-4 and IFN-γ, respectively (BD Biosciences). For analysis of Th17 polarization, 5 × 10^3^ moDCs pulsed for 24 h were cultured with 2 × 10^4^ allogenic memory CD4^+^ T cells for 6 d in the presence of *Staphylococcal enterotoxin B* (10 pg/mL). On d 6, supernatants were harvested and analyzed for IL-17A by ELISA (eBioscience). For blocking experiments, moDC–T cell cocultures were pre-incubated for 15 min with 10 μg/mL neutralizing PGE_2_ antibody (2B5; Cayman Chemical), 10 μM EP2 (AH6809; Cayman Chemical), 10 μM EP4 (AH23848; Cayman Chemical) receptor antagonist, 10 μg/mL anti-OX40L antibody (Clone 159403; R&D Systems), or IgG1 control antibody (clone P3.6.2.8.1; eBioscience).

### Detection of ROS

Detection of ROS was performed according to a published protocol (http://www.bio-protocol.org/e313) with minor modifications. In brief, after 6 h or 1 h of stimulation of moDCs or BMDCs, respectively, the cells were harvested, washed using 1% FCS RPMI, and resuspended in 50 μl containing 10 μM CM-H_2_DCFDA (C6827; Invitrogen), followed by an incubation at 37°C for 30 min. Prior to sample measurement, 7AAD was added. ROS levels were quantified by flow cytometry.

### Western blot

moDCs were harvested after 8 h of stimulation. Then, cells were washed twice with PBS before being lysed in EBSB buffer (8% [w/v] glycerol, 3% [w/v] SDS, and 100 mM Tris–HCl [pH 6.8]). Lysates were immediately boiled for 5 min, and their protein content was determined using a bicinchoninic acid protein assay kit (Thermo-Scientific, Waltham, MA). Proteins were separated by SDS-PAGE, followed by transfer to a PVDF membrane. Membranes were blocked for 1 h at room temperature in TTBS buffer (20 mM Tris–HCl [pH 7.6], 137 mM NaCl, and 0.25% [v/v] Tween 20) containing 5% (w/v) fat-free milk and incubated overnight with primary antibodies. The primary antibodies used were COX-1 (Cell Signalling Technology, Danvers, MA), COX-2 (Cell Signalling Technology), and actin (Merck). The membranes were then washed in TTBS buffer and incubated with horseradish peroxidase-conjugated secondary antibodies for 1 h at room temperature. After washing, blots were developed using enhanced chemiluminescence.

### Syk and ERK phosphorylation

For detection of phosphorylation of Syk (pSyk) and ERK (pERK), 2.5 × 10^4^ immature moDCs were seeded overnight in a 96-well flat-bottom plate. moDCs were stimulated with SEA (50 μg/ml), SEAΔα-1/ω-1 (50 μg/mL), or ω-1 (500 ng/mL) in the presence or absence of blocking antibodies or inhibitors (R406, anti-MR, anti–Dectin-1, anti–Dectin-2, combination of anti–Dectin-1 and anti–Dectin-2 or IgG1 and IgG2 control antibodies) for indicated periods, and the moDCs were fixed for 15 min with 4% ultrapure formaldehyde (Polysciences, Warrington, PA) directly in the plate. The cells were harvested and washed first with PBS and then with 0.5% of saponin for permeabilization. Cell were intracellularly stained with anti–phospo-Try525/526 Syk (clone C87C1) and anti–phospo-p44/42 MAPK (Erk1/2) (clone E10) (both Cell Signalling Technology). Following 2-h incubation at room temperature, cells were washed with 0.5% of saponin, and Syk and ERK phosphorylation was determined by flow cytometry.

### cPLA_2_ activity

cPLA_2_ activity was determined according to the manufacturer’s recommendation (Cayman Chemical). Briefly, 1 × 10^6^ moDCs stimulated for 8 h with indicated reagents were harvested. moDCs were lysed with lysis buffer (containing 50 mM Hepes, pH 7.4, 1 mM EDTA, NP-40, protease and phosphatase inhibitors) followed by 4 rounds of sonication for 10 s. The cells were then concentrated using a 30 KDa Amicon filter (Merck). To 10 μl cell lysate, 200 μl substrate solution was added to initiate the reaction, and the plate was briefly shaken and incubated for 1 h at room temperature. To stop the reaction, 10 μl DTNB/EGTA was added and the plate was briefly shaken, followed by 5-min incubation at room temperature. The cPLA_2_ activity was measured using a plate reader with absorbance of 405 nm.

### Antigen binding and uptake by DCs

SEA was fluorescently labelled with PF-647 using promofluor labelling kit (Promokine, Heidelberg, Germany) according to the manufacturer’s recommendations. Approximately 2 × 10^4^ immature moDCs or BMDCs per well were seeded in a flat-bottom 96-well plate. Where indicated, cells were pre-incubated with 20 μg/mL of anti-MR, anti–DC-SIGN, anti–Dectin-1, anti–Dectin-2, or control antibodies at 37°C for 45 min. Subsequently, cells were incubated with 2 μg/mL PF-647–labelled SEA at 37°C for 45 min for testing both binding and uptake of the antigen. After 45 min, cells were washed with PBS followed by flow cytometry measurement.

### LC-MS/MS analysis of PUFAs and LMs

Twenty μl of SEA or supernatants from each condition were collected at indicated time points after stimulation and stored at −80°C until analysis. A volume of 10 μl sample was mixed with 28.4 μL methanol (MeOH) and 1.6 μL of internal standard (containing Leukotriene B4-d4, 15-HETE-d8, PGE_2_-d4, and DHA-d5 at a concentration of 50 ng/mL in MeOH). The samples were subsequently kept at −20°C for 10 min for completion of protein precipitation, followed by centrifugation for 10 min, 16,000 × *g* at 4°C. Subsequently, samples were diluted 1:1 with water and transferred into auto-sampler vials. LC-MS/MS analysis using a QTrap 6500 (Sciex, the Netherlands) was carried out as described previously [61,62].

### BMDC culture and adoptive transfer

BMDCs were generated from indicated WT and KO mice as described previously [63]. Bone marrow cells were differentiated for 8 d in the presence of rGM-CSF (20 ng/ml) (PeproTech, Rocky Hill, NJ) in RPMI-1640 medium containing 5% FCS, 100 U/ml penicillin-streptomycin, and 2 mM glutamine. On d 3 and 6, culture medium was refreshed. Nonadherent cells, containing routinely >80% CD11c^+^MHC-II^+^ DCs were used for various assays, including ROS production, SEA binding/uptake, OX40L expression, and Th2-priming experiments in vivo. OX40L expression was assessed 18 h after stimulation with SEAΔα-1/ω-1 (50 μg/mL) by flow cytometry following surface staining with anti-OX40L (clone RM134L; Biolegend). For in vivo experiments, BMDCs were stimulated with SEAΔα-1/ω-1 (50 μg/mL) for 18 h, washed 3 times in HBSS, and injected into WT recipient mice (400.000/footpad). Analysis of T-cell responses in draining popliteal LNs 7 d later was performed as described below.

### SEA, BMDC immunization, and *S*. *mansoni* infection

Mice were injected subcutaneously with SEA (20 μg), SEAΔα-1/ω-1 (20 μg), ω-1 (2 μg), or 400.000 SEAΔα-1/ω-1–pulsed BMDCs in the hind footpad. Seven d later, cells from both draining and nondraining lymph nodes were isolated and analyzed as described below. For *S*. *mansoni* infection, mice were infected with 100 cercariae from a Brazilian strain of *S*. *mansoni* obtained from our in-house cycle of infected *Biomphalaria glabrata* snails (also of Brazilian origin). Mice were killed after 8 wk of infection. Liver samples were fixed in 4% buffered formalin and embedded in paraffin. Sections (4 μm) were stained with Masson blue and examined microscopically (Axioskop; Zeiss, Oberkochen, Germany) for measuring the diameters to calculate the size of spherical granulomas. To determine the parasite burden, pieces of weighed liver and intestine samples from individual mice were digested in 4% KOH at 37°C for 4 h. After centrifugation, the released eggs were microscopically counted. The absolute number of eggs in the liver and intestine was then calculated in accordance with the total organ weight. Worm burden was calculated as adult worm recovery after portal perfusion and microscopic examination of livers and intestines.

### Analyses of murine T-cell responses

Antigen-specific recall responses were determined by culturing 3 × 10^5^ LN or spleen cells per well in 96-well round-bottom plates in 200 μl complete medium (RPMI containing 10% fetal calf serum, 100 U/ml penicillin/streptomycin, and 2 mM l-glutamine) in the presence of 20 μg/ml SEA or 1 μg/ml anti-CD3/CD28 antibody (eBioscience). 2.5 μg/ml IL-4R blocking antibody (M1) was added to the cultures to retain IL-4 in culture supernatants. After 72 h, culture supernatants were stored for cytokine determination. Cell culture supernatants were analyzed for cytokines using the Cytokine Bead Array (BD) or the mouse Ready-Set-Go ELISA kits (eBioscience) according to the manufacturer’s recommendation. Samples were analyzed on a BD Canto II Flow Cytometer and Sunrise ELISA microplate reader (Tecan, Morrisville, NC), respectively. Alternatively, assessment of cytokine production by intracellular staining of T cells from LNs was determined after polyclonal restimulation in 96-well round-bottom plates for 5 h with phorbol 12-myristate 13-acetate (PMA; 50 ng/ml) and ionomycin (1 μg/ml) in the presence of brefeldin A (10 μg/ml; all from Sigma-Aldrich) for the last 3 h. Afterwards, cells were fixed with 4% PFA and subsequently stained in 0.5% saponin with antibodies against the following antigens: CD44 (IM7), IL-4 (11B11), IFN-γ (XMG1.2), IL-13 (eBio13A), IL-17A (TC11-18H10.1), and CD4 (RM4-5) (all BD Bioscience or Biolegend). Samples were analyzed on a BD Canto II Flow Cytometer.

### Statistical analysis

The heatmap was generated using Microsoft Excel. Data were analyzed for normal distribution (Shapiro-Wilk normality test) and statistical significance using two-way ANOVA test, two-sided paired Student *t* test, or unpaired Student *t* test. Statistical analysis was performed using GraphPad Prism version 6.00 (GraphPad Software, La Jolla, CA) for Windows.

## Supporting information

S1 Fig LM composition of SEA and in supernatants of SEA-conditioned moDCs. (A) Concentration of 22 LMs, out of 55 potentially detectable LMs, that are present in SEA from *S*. *mansoni* as determined by LC-MS/MS. LMs are ordered according to abundance, and concentrations are determined based on internal standards. (B) moDCs were pulsed with SEA or ω-1 in combination with LPS, after which supernatants were collected at 0, 6, 12, and 24 h after stimulation. Relative amounts of PUFAs and LMs detected by LC-MS/MS in supernatants are shown in a heat map. Data represent an average of 3 independent experiments. Color coding is based on relative abundance of each lipid in comparison to other time points or stimulations. (C) As in panel B but without LPS. Data represent 1 of 2 independent experiments. (D) moDCs were pulsed with indicated reagents after which supernatants were collected at 24 h after stimulation. Relative amounts of PGE_2_ detected by LC-MS/MS in supernatants are shown. Bar graphs represent means ± SEM of 3 independent experiments. *P < 0.05, for significant differences with the control conditions based on unpaired Student *t* test. Underlying data can be found in S1 Data. ω-1, omega-1; LC-MS/MS, liquid chromatography tandem mass spectrometry; LM, lipid mediator; LPS, lipopolysaccharide; moDC, monocyte-derived DC; PGE_2_, prostaglandin E_2_; PUFA, polyunsaturated fatty acid; SEA, soluble egg antigen.
(TIF)

S2 Fig OX40L is induced by SEA via PGE_2_ signaling and is required for Th2 induction independently of LPS. (A) T-cell polarization assay as described in main figures. (B, C) moDCs were stimulated as indicated for 48 h in the presence or absence of neutralizing anti-PGE_2_ antibody after which expression of OX40L was analyzed by flow cytometry. The fold change based on geometric mean fluorescence is shown relative to LPS, which is set to 1 (dashed line). (D) T-cell polarization assay as described in main figures. Neutralizing OX40L antibody was added during the DC–T cell coculture. Bar graphs represent means ± SEM of at least 3 independent experiments. “*” and “#”: *P* < 0.05 for significant differences with the control conditions (*) or between-test conditions (#) based on unpaired analysis (unpaired Student *t* test). Underlying data can be found in S1 Data. LPS, lipopolysaccharide; moDC, monocyte-derived DC; OX40L, OX40 ligand; PGE_2_, prostaglandin E_2_; SEA, soluble egg antigen; Th2, T helper 2.
(TIF)

S3 Fig SEA-driven PGE2 synthesis is similar between IL-4– and IL-13–cultured human moDCs. (A) CD1a expression was assessed as a marker for moDC differentiation of monocytes that were differentiated for 6 d in the presence of GM-CSF plus IL-4 or GM-CSF plus IL-13. Representative graphs of 2 independent experiments are shown. (B) PGE_2_ production by IL-4– or IL-13–cultured moDCs in response to SEAΔα-1/ω-1 16 h after stimulation. Bar graphs represent means ± SEM of 2 independent experiments. Underlying data can be found in S1 Data. ω-1, omega-1; GM-CSF, granulocyte-macrophage colony-stimulating factor; IL-4, interleukin 4; LPS, lipopolysaccharide; moDC, monocyte-derived DC; PGE_2_, prostaglandin E_2_; SEA, soluble egg antigen.
(TIF)

S4 Fig SEA does not condition human moDCs for priming of Th17 responses. (A) IL-23 levels were determined in supernatants of moDC cultures that were stimulated with indicated reagents for 40 h. (B) IL-17 production was assessed in culture supernatants of T cells that were cultured with moDCs that were stimulated with indicated reagents. Zymosan was taken along as positive control stimulus for Th17 induction. Bar graphs represent means ± SEM of at least 4 independent experiments. Underlying data can be found in S1 Data. IL-23, interleukin 23; moDC, monocyte-derived DC; O.p., outpositive; SEA, soluble egg antigen; Th17, T helper 17.
(TIF)

S5 Fig BMDCs require Syk expression for Th2 priming by *S*. *mansoni* egg antigens in vivo. (A) BMDCs cultured from BM from CD11c^WT^ or CD11c^ΔSyk^ mice were pulsed overnight with SEAΔα-1/ω-1, injected into hind footpads after which CD4^+^ T-cell responses were analyzed as in Fig 6A. Representative flow cytometry plots of intracellular staining of CD4^+^ T cells are depicted, of which the data are enumerated in bar graphs representing mean ± SEM of 3 to 4 mice per group. (B) Ratio between percent IL-4– and IFN-γ–producing T cells as described in panel A. (C) SEA binding and uptake by indicated BMDCs was determined as in Fig 4F. (D) ROS production by indicated BMDCs was determined as described in Fig 5E 1 h after stimulation with SEAΔα-1/ω-1. (E) BMDCs were stimulated as indicated for 18 h after which expression of OX40L was analyzed by flow cytometry. Representative plots are depicted, of which the data are enumerated in bar graphs and shown as fold change relative to control condition, which is set to 1. (C–E) Bar graphs represent duplicates ± SEM of 2 independent experiments. “*” and “#”: *P* < 0.05; “**” and “##”: *P* < 0.01; “***” and “###”: *P* < 0.001 for significant differences with the control conditions (*) or between-test conditions (#) based on unpaired analysis (unpaired Student *t* test). Underlying data can be found in S1 Data. ω-1, omega-1; BMDC, bone marrow–derived DC; CD4, cluster of differentiation 4; H2-DCFDA, 2',7'-dichlorodihydrofluorescein diacetate; IFN-γ, interferon γ; IL-4, interleukin 4; ndLN, non-draining lymph node; OX40L, OX40 ligand; ROS, reactive oxygen species; SEA, soluble egg antigen; Syk, spleen tyrosine kinase; Th2, T helper2; WT, wild-type.
(TIF)

S6 Fig Analysis of Th17 responses by SEAΔα-1/ω-1–pulsed BMDCs induced in vivo. BMDCs cultured from BM from WT, Dectin-1^−/−^, or Dectin-2^−/−^ mice were pulsed overnight with SEAΔα-1/ω-1 and injected into hind footpads after which Th17 responses were analyzed as in Fig 6A. Representative flow cytometry plots of intracellular IL-17A staining of CD4^+^ T cells are depicted, of which the data are enumerated in bar graphs representing mean ± SEM of 2 independent experiments with 4 mice per group. “*” and “#”: *P* < 0.05 for significant differences with the control conditions (*) or between-test conditions (#) based on unpaired analysis (unpaired Student *t* test). Underlying data can be found in S1 Data. ω-1, omega-1; BMDC, bone marrow–derived DC; CD4, cluster of differentiation 4; IL-17A, interleukin 17A; SEA, soluble egg antigen; Th17, T helper 17; WT, wild-type.
(TIF)

S7 Fig Dectin-1 signaling plays a minor role in Th2 priming during *S*. *mansoni* infection. WT and Dectin-1^−/−^ mice were infected with *S*. *mansoni*. After 8 wk of infection, cells from spleens (A) or mLNs (B) were restimulated with SEA or anti-CD3/CD28 for 72 h, and cytokine levels were analyzed in supernatants by ELISA. Bars represent mean ± SEM of combined data of 3 independent experiments with 3 to 4 mice per group. (c) Granuloma sizes around eggs trapped in the liver of 8-week–infected mice were assessed in Masson blue–stained liver sections. Data are based on 10 mice per group. Number of worms (D) and liver and intestinal eggs (E) in mice infected with *S*. *mansoni* for 8 wk. *P < 0.05 for significant differences relative to the control mice based on unpaired analysis (unpaired Student *t* test). Underlying data can be found in S1 Data. CD3, cluster of differentiation 3; mLN, mesenteric lymph node; SEA, soluble egg antigen; Th2, T helper 2; WT, wild-type.
(TIF)

S1 Table LMs including PUFAs that were measured using LC-MS/MS. LC-MS/MS, liquid chromatography tandem mass spectrometry; LM, lipid mediator; PUFA, polyunsaturated fatty acid.
(DOCX)

S1 Data (XLSX)

## Abbreviations

13-HODE: 13-Hydroxyoctadecadienoic acid
ω-1: omega-1
AA: arachidonic acid
APC: antigen-presenting cell
BMDC: bone marrow–derived DC
CD4: cluster of differentiation 4
CLR: c-type lectin receptor
COX-1: cyclooxygenase 1
cPLA_2_: cytosolic phospholipase A_2_
DC: dendritic cell
DC-SIGN: dendritic cell-specific intercellular adhesion molecule-3-grabbing non-integrin
EP2/4: prostaglandin E_2_ receptor 2/4
ERK: extracellular signal-regulated kinase
FcγR: Fc gamma receptor
HETE: Hydroxyeicosatetraenoic acid
IL-4: interleukin 4
IFN-γ: interferon gamma
IsoP: isoprostane
ITAM: immunoreceptor tyrosine-based activation motif
LC-MS/MS: liquid chromatography tandem mass spectrometry
LM: lipid mediator
LN: lymph node
LPS: lipopolysaccharide
LXA_4_: lipoxin A_4_
mLN: mesenteric LN
moDC: monocyte-derived DC
MR: mannose receptor
NAC: *N*-acetyl-*L*-cysteine
OX40L: OX40 ligand
PAMP: pathogen associated molecular pattern
PG: prostaglandin
PGE_2_: prostaglandin E_2_
pLN: popliteal LN
PMA: phorbol 12-myristate 13-acetate
PRR: pattern-recognition receptor
PUFA: polyunsaturated fatty acid
ROS: reactive oxygen species
SEA: soluble egg antigen
Syk: spleen tyrosine kinase
Th2: T helper 2
Treg: regulatory T cell
TSLP: thymic stromal lymphopoietin
WT: wild-type
