# Supplementary figures and images for: Dectin-1/2–induced autocrine PGE2 signaling licenses dendritic cells to prime Th2 responses
Source: PLoS Biol. 2018 Apr 18;16(4):e2005504. doi: 10.1371/journal.pbio.2005504 (PMC5927467; doi:10.1371/journal.pbio.2005504)

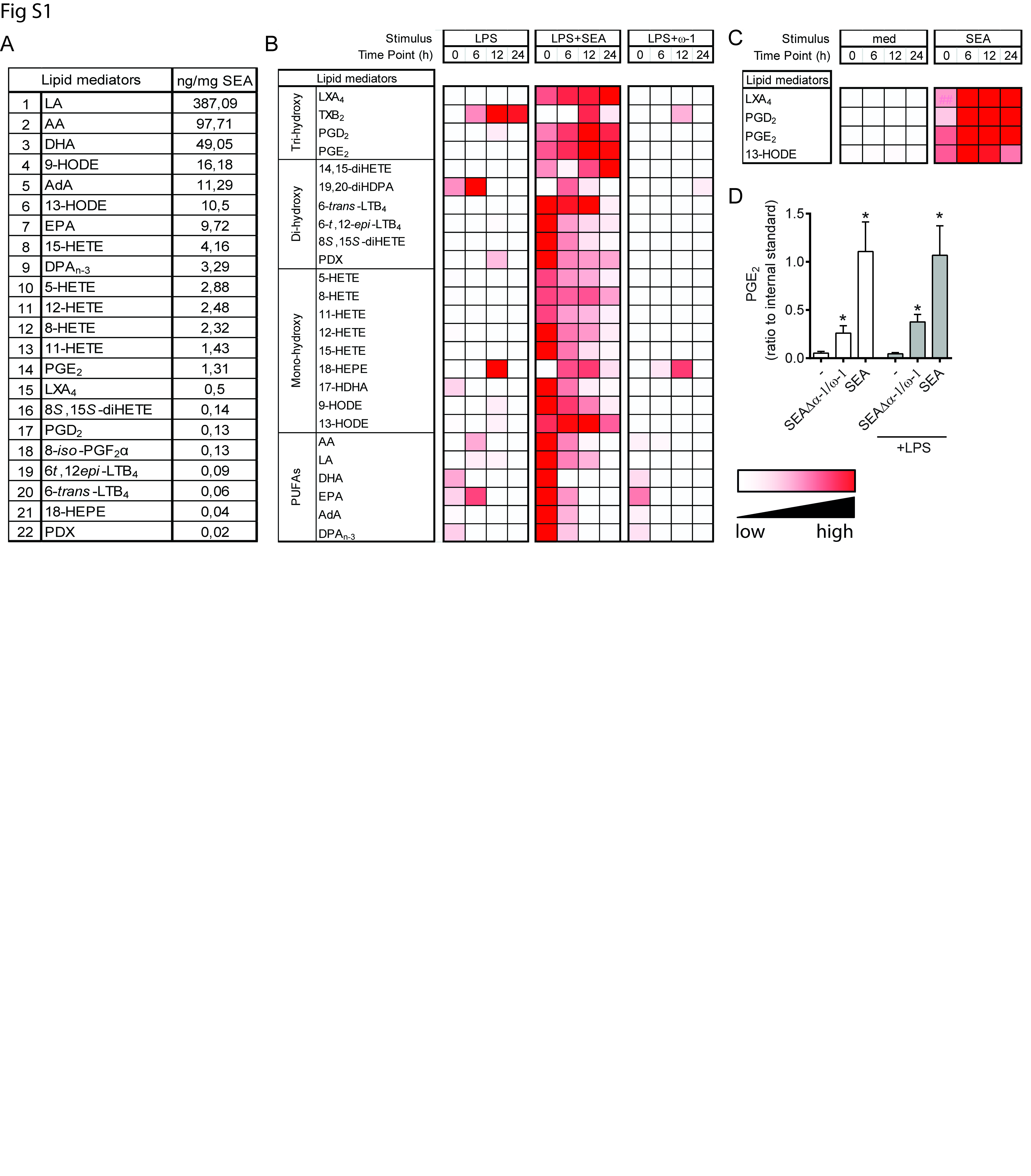

Supplement: S1 Fig — (A) Concentration of 22 LMs, out of 55 potentially detectable LMs, that are present in SEA from S. mansoni as determined by LC-MS/MS. LMs are ordered according to abundance, and concentrations are determined based on internal standards. (B) moDCs were pulsed with SEA or ω-1 in combination with LPS, after which supernatants were collected at 0, 6, 12, and 24 h after stimulation. Relative amounts of PUFAs and LMs detected by LC-MS/MS in supernatants are shown in a heat map. Data represent an average of 3 independent experiments. Color coding is based on relative abundance of each lipid in comparison to other time points or stimulations. (C) As in panel B but without LPS. Data represent 1 of 2 independent experiments. (D) moDCs were pulsed with indicated reagents after which supernatants were collected at 24 h after stimulation. Relative amounts of PGE2 detected by LC-MS/MS in supernatants are shown. Bar graphs represent means ± SEM of 3 independent experiments. *P < 0.05, for significant differences with the control conditions based on unpaired Student t test. Underlying data can be found in S1 Data. ω-1, omega-1; LC-MS/MS, liquid chromatography tandem mass spectrometry; LM, lipid mediator; LPS, lipopolysaccharide; moDC, monocyte-derived DC; PGE2, prostaglandin E2; PUFA, polyunsaturated fatty acid; SEA, soluble egg antigen. (TIF) [file pbio.2005504.s001.tif]

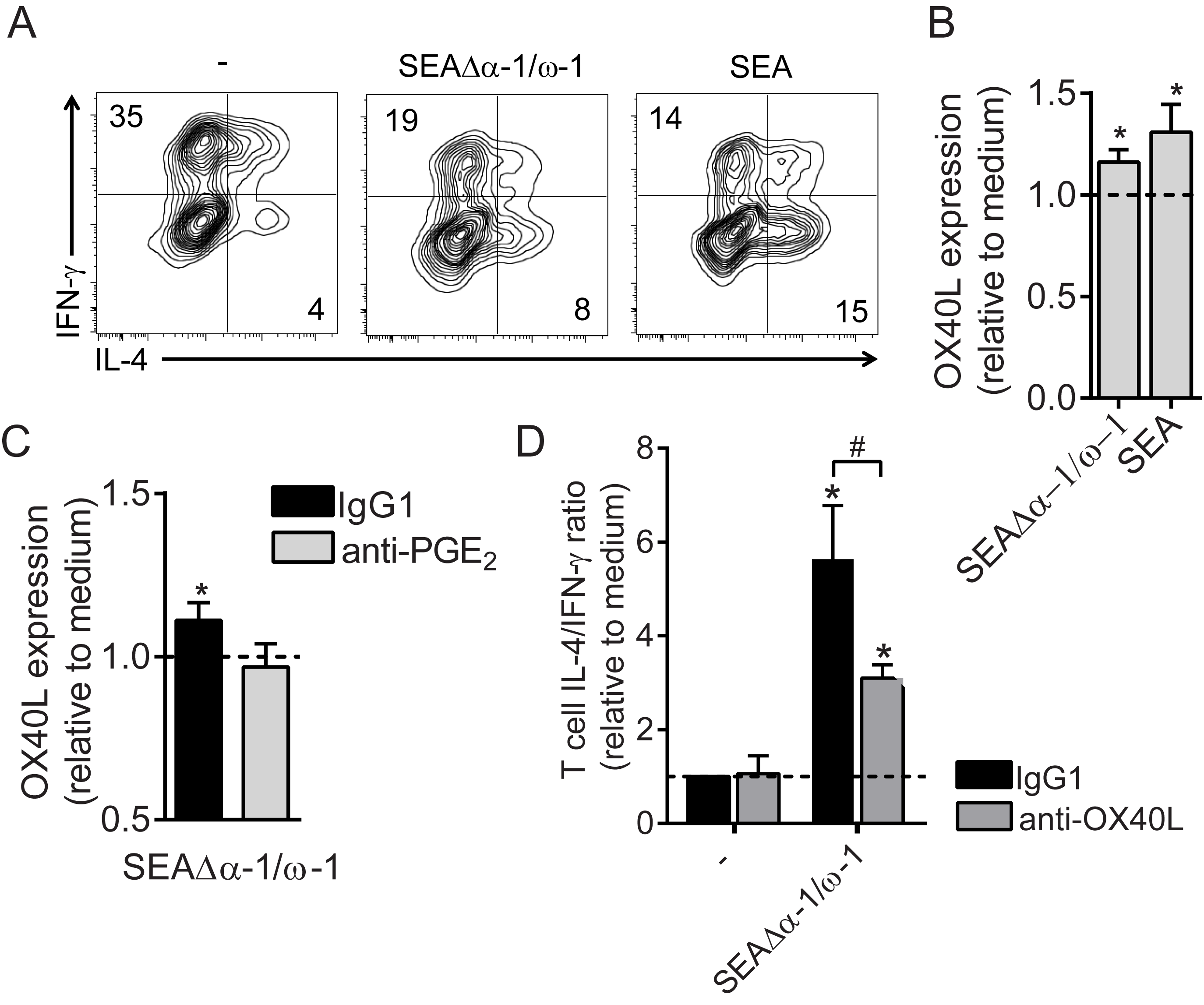

Supplement: S2 Fig — (A) T-cell polarization assay as described in main figures. (B, C) moDCs were stimulated as indicated for 48 h in the presence or absence of neutralizing anti-PGE2 antibody after which expression of OX40L was analyzed by flow cytometry. The fold change based on geometric mean fluorescence is shown relative to LPS, which is set to 1 (dashed line). (D) T-cell polarization assay as described in main figures. Neutralizing OX40L antibody was added during the DC–T cell coculture. Bar graphs represent means ± SEM of at least 3 independent experiments. “*” and “#”: P < 0.05 for significant differences with the control conditions (*) or between-test conditions (#) based on unpaired analysis (unpaired Student t test). Underlying data can be found in S1 Data. LPS, lipopolysaccharide; moDC, monocyte-derived DC; OX40L, OX40 ligand; PGE2, prostaglandin E2; SEA, soluble egg antigen; Th2, T helper 2. (TIF) [file pbio.2005504.s002.tif]

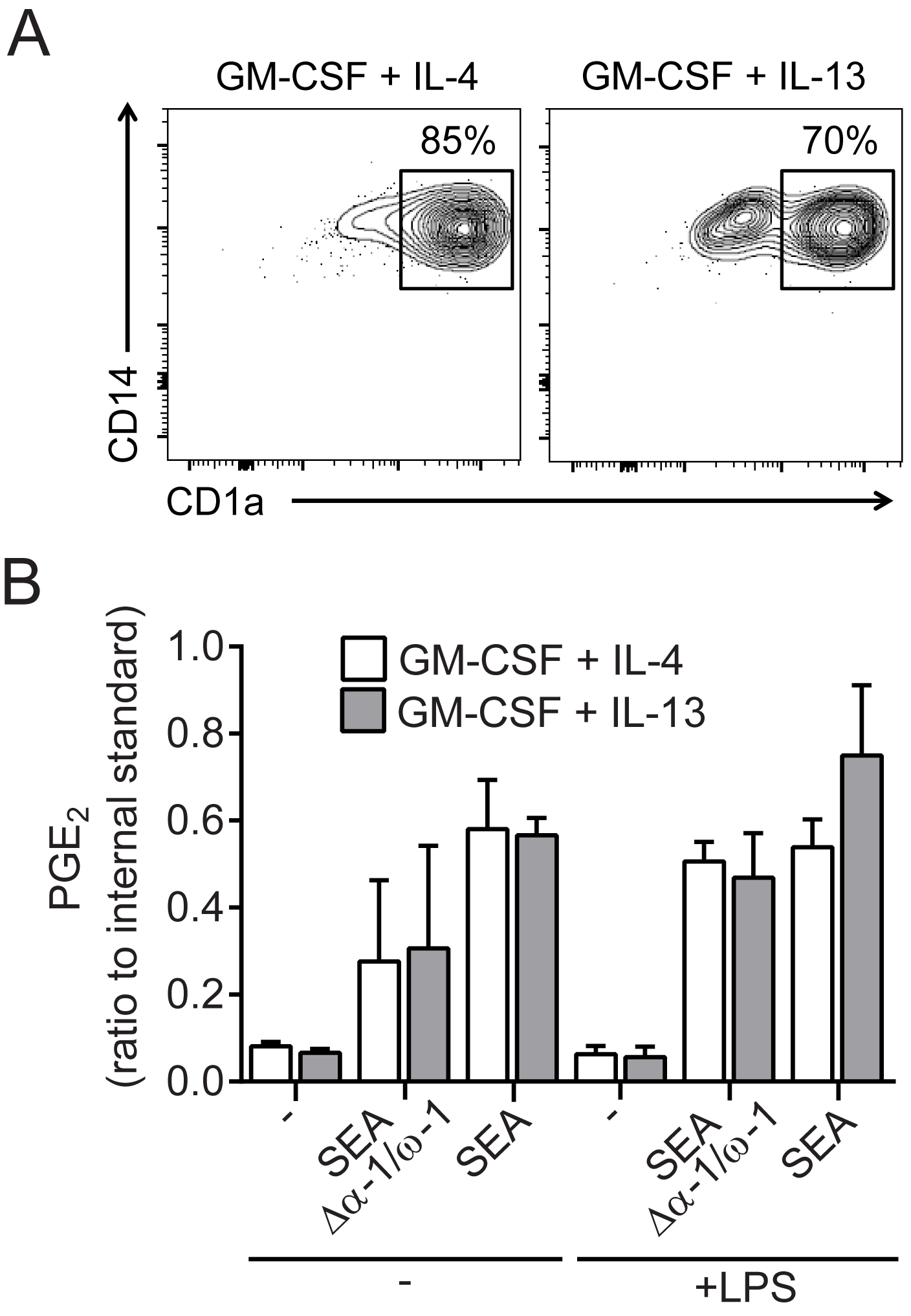

Supplement: S3 Fig — (A) CD1a expression was assessed as a marker for moDC differentiation of monocytes that were differentiated for 6 d in the presence of GM-CSF plus IL-4 or GM-CSF plus IL-13. Representative graphs of 2 independent experiments are shown. (B) PGE2 production by IL-4– or IL-13–cultured moDCs in response to SEAΔα-1/ω-1 16 h after stimulation. Bar graphs represent means ± SEM of 2 independent experiments. Underlying data can be found in S1 Data. ω-1, omega-1; GM-CSF, granulocyte-macrophage colony-stimulating factor; IL-4, interleukin 4; LPS, lipopolysaccharide; moDC, monocyte-derived DC; PGE2, prostaglandin E2; SEA, soluble egg antigen. (TIF) [file pbio.2005504.s003.tif]

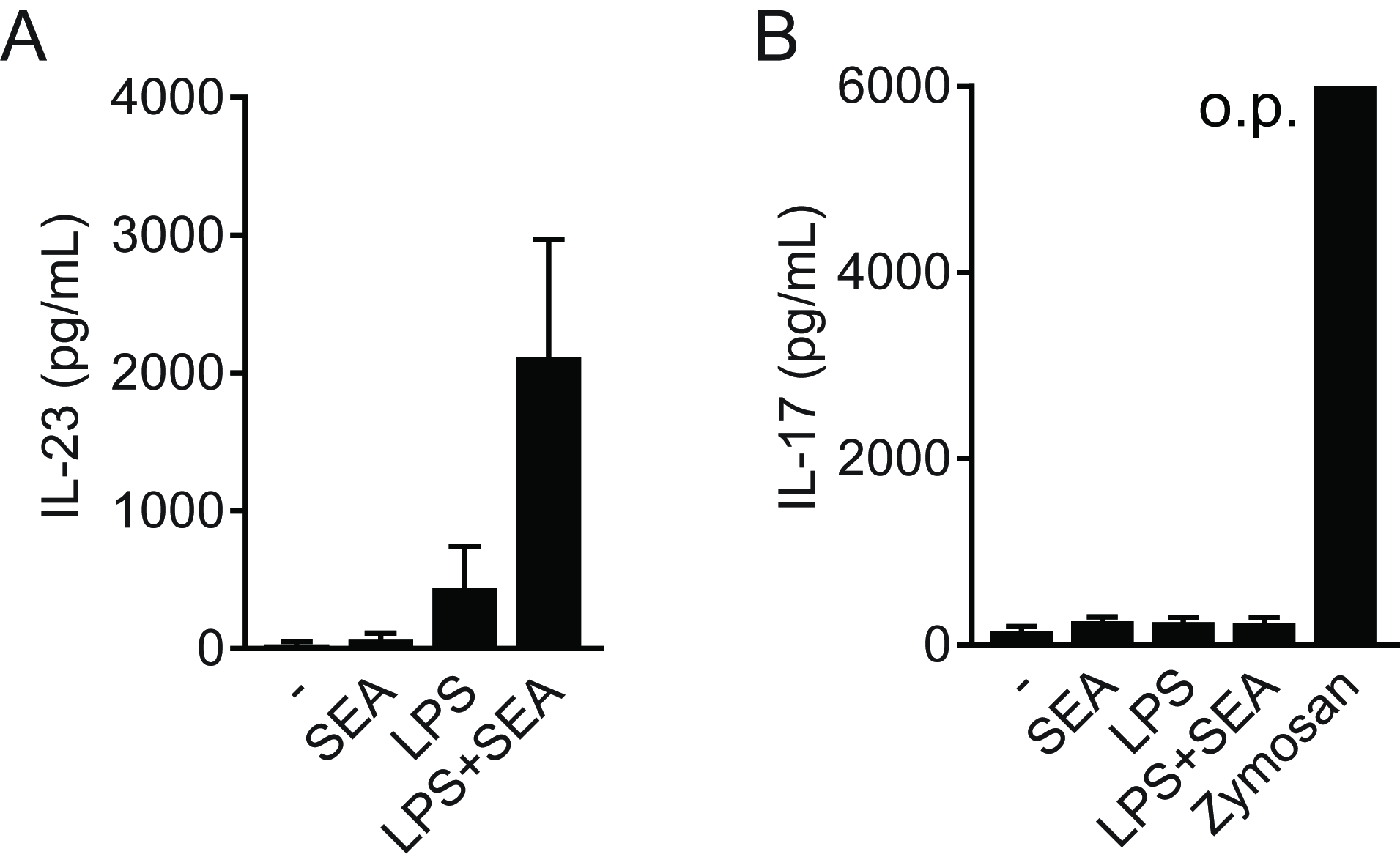

Supplement: S4 Fig — (A) IL-23 levels were determined in supernatants of moDC cultures that were stimulated with indicated reagents for 40 h. (B) IL-17 production was assessed in culture supernatants of T cells that were cultured with moDCs that were stimulated with indicated reagents. Zymosan was taken along as positive control stimulus for Th17 induction. Bar graphs represent means ± SEM of at least 4 independent experiments. Underlying data can be found in S1 Data. IL-23, interleukin 23; moDC, monocyte-derived DC; O.p., outpositive; SEA, soluble egg antigen; Th17, T helper 17. (TIF) [file pbio.2005504.s004.tif]

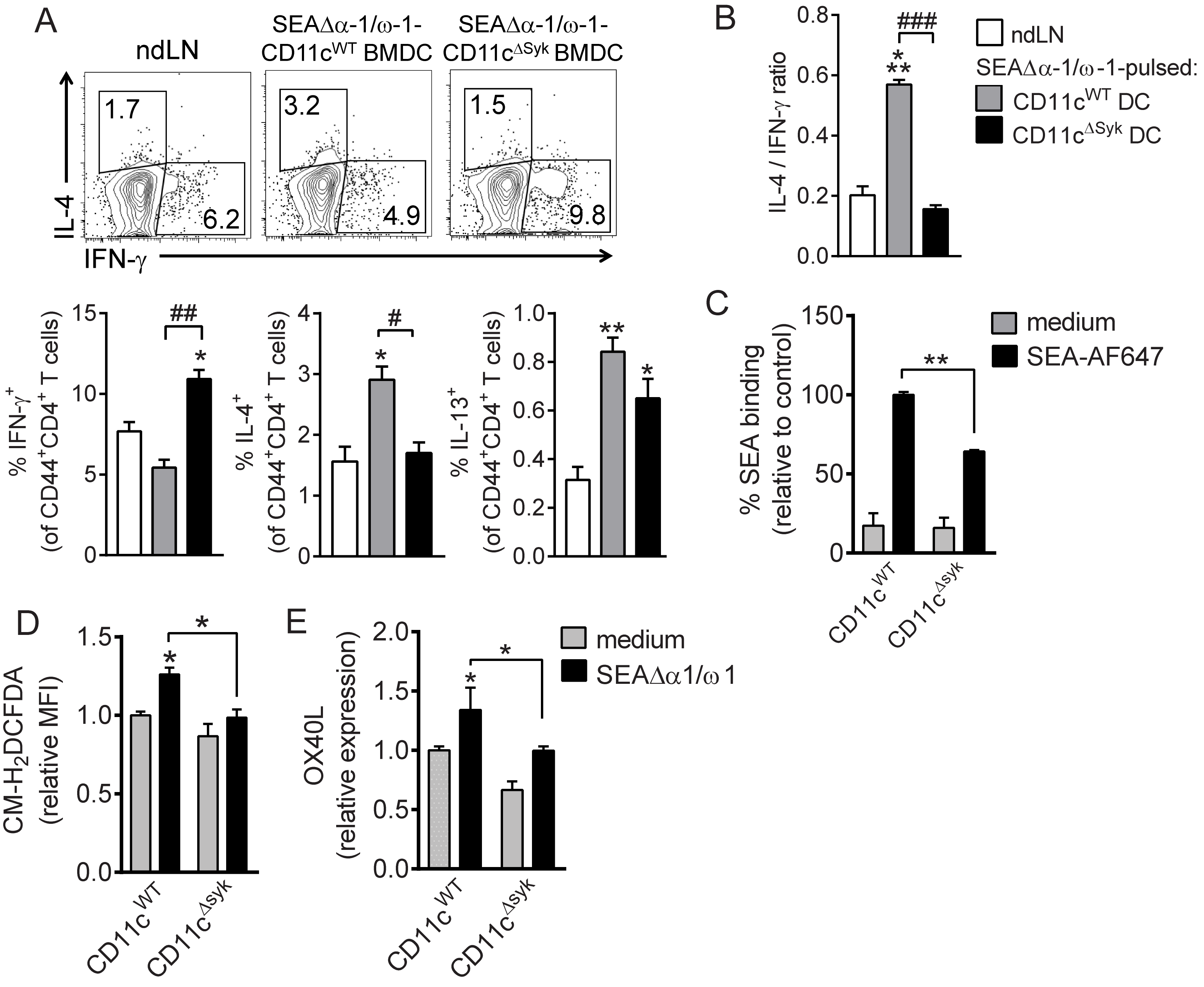

Supplement: S5 Fig — (A) BMDCs cultured from BM from CD11cWT or CD11cΔSyk mice were pulsed overnight with SEAΔα-1/ω-1, injected into hind footpads after which CD4+ T-cell responses were analyzed as in Fig 6A. Representative flow cytometry plots of intracellular staining of CD4+ T cells are depicted, of which the data are enumerated in bar graphs representing mean ± SEM of 3 to 4 mice per group. (B) Ratio between percent IL-4– and IFN-γ–producing T cells as described in panel A. (C) SEA binding and uptake by indicated BMDCs was determined as in Fig 4F. (D) ROS production by indicated BMDCs was determined as described in Fig 5E 1 h after stimulation with SEAΔα-1/ω-1. (E) BMDCs were stimulated as indicated for 18 h after which expression of OX40L was analyzed by flow cytometry. Representative plots are depicted, of which the data are enumerated in bar graphs and shown as fold change relative to control condition, which is set to 1. (C–E) Bar graphs represent duplicates ± SEM of 2 independent experiments. “*” and “#”: P < 0.05; “**” and “##”: P < 0.01; “***” and “###”: P < 0.001 for significant differences with the control conditions (*) or between-test conditions (#) based on unpaired analysis (unpaired Student t test). Underlying data can be found in S1 Data. ω-1, omega-1; BMDC, bone marrow–derived DC; CD4, cluster of differentiation 4; H2-DCFDA, 2',7'-dichlorodihydrofluorescein diacetate; IFN-γ, interferon γ; IL-4, interleukin 4; ndLN, non-draining lymph node; OX40L, OX40 ligand; ROS, reactive oxygen species; SEA, soluble egg antigen; Syk, spleen tyrosine kinase; Th2, T helper2; WT, wild-type. (TIF) [file pbio.2005504.s005.tif]

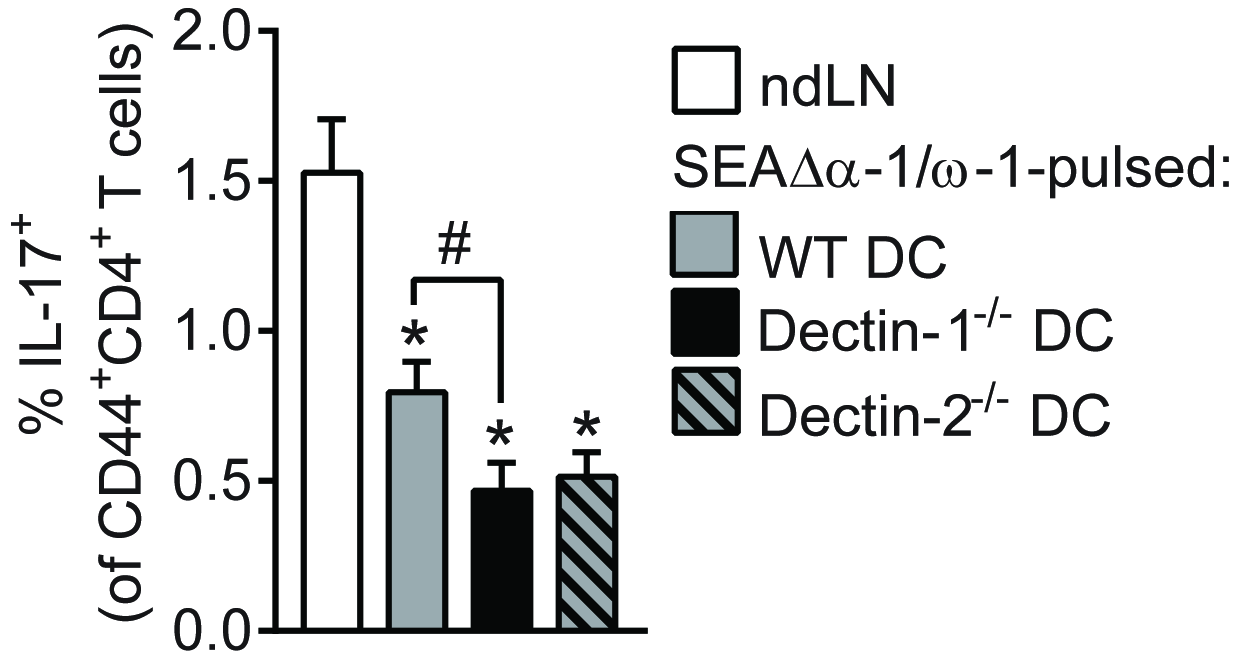

Supplement: S6 Fig — BMDCs cultured from BM from WT, Dectin-1−/−, or Dectin-2−/− mice were pulsed overnight with SEAΔα-1/ω-1 and injected into hind footpads after which Th17 responses were analyzed as in Fig 6A. Representative flow cytometry plots of intracellular IL-17A staining of CD4+ T cells are depicted, of which the data are enumerated in bar graphs representing mean ± SEM of 2 independent experiments with 4 mice per group. “*” and “#”: P < 0.05 for significant differences with the control conditions (*) or between-test conditions (#) based on unpaired analysis (unpaired Student t test). Underlying data can be found in S1 Data. ω-1, omega-1; BMDC, bone marrow–derived DC; CD4, cluster of differentiation 4; IL-17A, interleukin 17A; SEA, soluble egg antigen; Th17, T helper 17; WT, wild-type. (TIF) [file pbio.2005504.s006.tif]

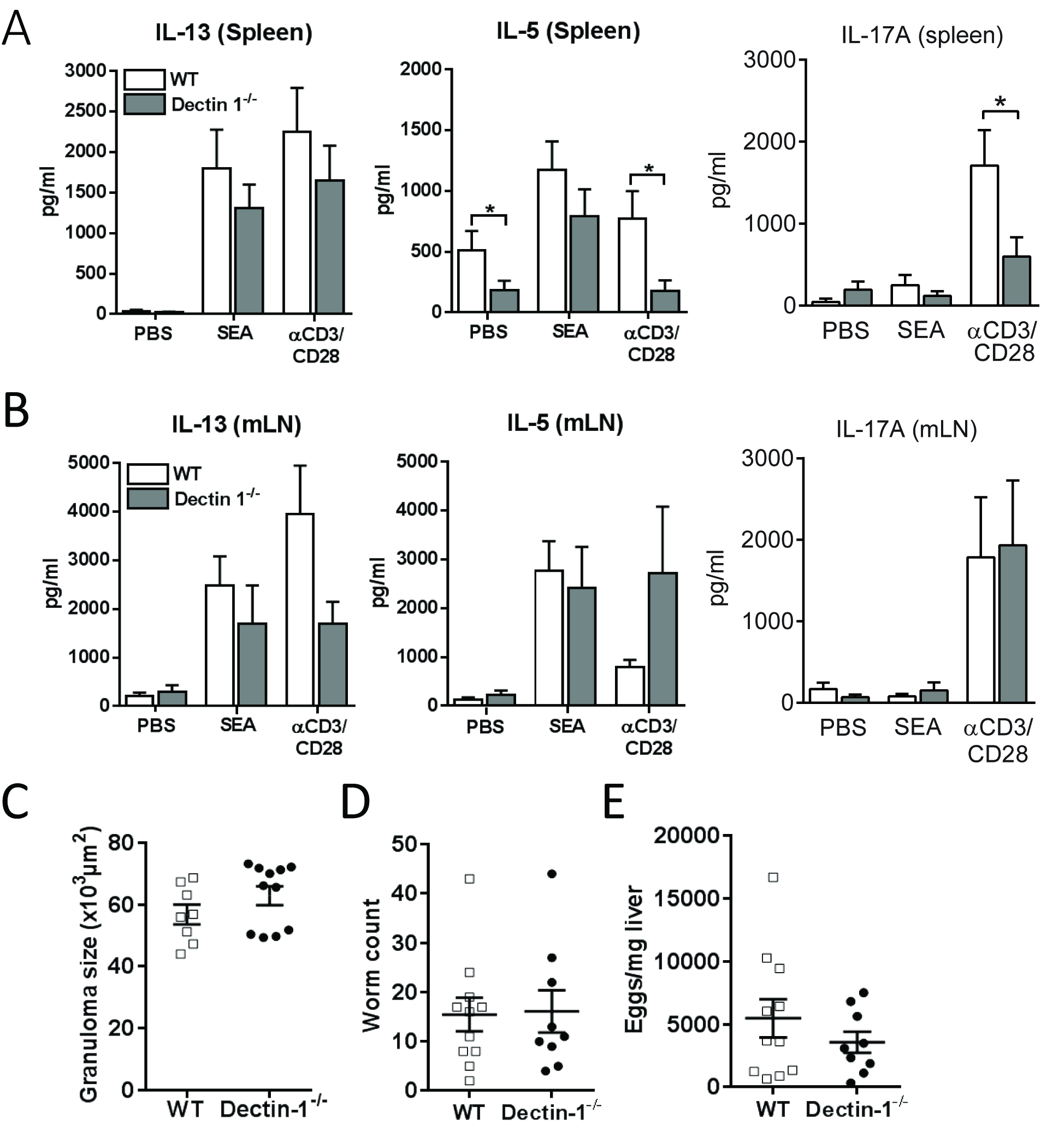

Supplement: S7 Fig — WT and Dectin-1−/− mice were infected with S. mansoni. After 8 wk of infection, cells from spleens (A) or mLNs (B) were restimulated with SEA or anti-CD3/CD28 for 72 h, and cytokine levels were analyzed in supernatants by ELISA. Bars represent mean ± SEM of combined data of 3 independent experiments with 3 to 4 mice per group. (c) Granuloma sizes around eggs trapped in the liver of 8-week–infected mice were assessed in Masson blue–stained liver sections. Data are based on 10 mice per group. Number of worms (D) and liver and intestinal eggs (E) in mice infected with S. mansoni for 8 wk. *P < 0.05 for significant differences relative to the control mice based on unpaired analysis (unpaired Student t test). Underlying data can be found in S1 Data. CD3, cluster of differentiation 3; mLN, mesenteric lymph node; SEA, soluble egg antigen; Th2, T helper 2; WT, wild-type. (TIF) [file pbio.2005504.s007.tif]
