# Supplementary material for: Dectin-1/2–induced autocrine PGE2 signaling licenses dendritic cells to prime Th2 responses
Source: PLoS Biol. 2018 Apr 18;16(4):e2005504. doi: 10.1371/journal.pbio.2005504 (PMC5927467; doi:10.1371/journal.pbio.2005504)
Supplement: S1 Table — LC-MS/MS, liquid chromatography tandem mass spectrometry; LM, lipid mediator; PUFA, polyunsaturated fatty acid. (DOCX) [file pbio.2005504.s008.docx]

Supplementary Table 1 is Lipid Mediators (LM) including Polyunsaturated Fatty Acids (PUFAs) that were measured using LC-MS/MS

| LMs analysed by GC-MS/MS | | | | | |
| --- | --- | --- | --- | --- | --- |
| 1 | 10-HDHA | 10-hydroxy Docosahexaenoic acid | 29 | 8*S*,15*S*-diHETE | 18S, 15S-dihydroxyeicosatetraenoic acid |
| 2 | 11-HETE | 11-hydroxyeicosatetraenoic acid | 30 | 9-HoDE | 9-hydroxyoctadecadienoic acid |
| 3 | 12-HETE | 12-hydroxyeicosatetraenoic acid | 31 | 9-HoTrE | 9-hydroxyoctadecatrienoic acid |
| 4 | 13,14dihydro-15-keto-PGE_2_ | 13, 14dihidro-15-keto-Prostaglandin E2 | 32 | AA | Arachidonic acid |
| 5 | 13,14dihydro-15-keto-PGF_2_α | 13, 14dihidro-15-keto-Prostaglandin F2α | 33 | AdA | Adrenic acid |
| 6 | 13-HoDE | 13-hydroxyoctadecadienoic acid | 34 | ALA | α-linolenic acid |
| 7 | 13-HoTrE | 13-hydroxyoctadecatrienoic acid | 35 | AT-LXA_4_ | AT-Lipoxin A4 |
| 8 | 14,15-diHETE | 14, 15-dihydroxyeicosatetraenoic acid | 36 | AT-RvD1 | AT-Resolvin D1 |
| 9 | 15-HEPE | 15-hydroperoxyeicosapentanoic acids | 37 | DHA | Docosahexaenoic |
| 10 | 15-HETE | 15-hydroxyeicosatetraenoic acid | 38 | DPA_n-3_ | Docosapentaenoic acid n-3 |
| 11 | 15-Keto-PGE_2_ | 15-Keto-Prostaglandin E2 | 39 | EPA | Eicosapentaenoic acid |
| 12 | 17-HDHA | 17-hydroxy Docosahexaenoic acid | 40 | LA | Linoleic acid |
| 13 | 17-OH-DH-HETE | 17-OH-DH- hydroxyeicosatetraenoic acid | 41 | LTB_4_ | Leukotriene B4 |
| 14 | 18-HEPE | 18-hydroperoxyeicosapentanoic acids | 42 | LTD_4_ | Leukotriene D4 |
| 15 | 18*R*-RvE3 | 18R-Resolvin E3 | 43 | LTE_4_ | Leukotriene E4 |
| 16 | 18*S*-RvE3 | 18S-Resolvin E3 | 44 | LXA_4_ | Lipoxin A4 |
| 17 | 19,20-diHDPA | 19, 20-dihydroxydocosapentanoic acid | 45 | MaR1_2 | Maresin 1_2 |
| 18 | 20-OH-LTB_4_ | 20-OH-Leukotrine B4 | 46 | PDX | Protectin DX |
| 19 | 5,15-diHETE | 5, 15-dihydroxyeicosatetraenoic acid | 47 | PGD_2_ | Prostaglandin D2 |
| 20 | 5-HETE | 5-hydroxyeicosatetraenoic acid | 48 | PGE_2_ | Prostaglandin E2 |
| 21 | 6*t*,12*epi*-LTB_4_ | 6t, 12epi-Leukotrine B4 | 49 | PGF_2_α | Prostaglandin F2a |
| 22 | 6-*trans*-LTB_4_ | 6-trans-Leukotrine B4 | 50 | PGJ_2_ | Prostaglandin J2 |
| 23 | 7,17-diHDPA | 7, 17-dihydroxydocosapentanoic acid | 51 | RvD1 | Resolvin D1 |
| 24 | 7-HDHA | 7-hydroxy Docosahexaenoic acid | 52 | RvD2 | Resolvin D2 |
| 25 | 7*S*-MaR1 | 7S-Maresin 1 | 53 | RvE1 | Resolvin E1 |
| 26 | 8-HETE | 8-hydroxyeicosatetraenoic acid | 54 | RvE2 | Resolvin E2 |
| 27 | 8-iso-PGE_2_ | 8-iso-Prostaglandin E2 | 55 | TxB2 | Thromboxane-B2 |
| 28 | 8-iso-PGF_2_α | 8-iso-Prostaglandin F2α |  |  |  |
